# Metagenomics reveals spatial variation in cyanobacterial composition, function, and biosynthetic potential in the Winam Gulf, Lake Victoria, Kenya

**DOI:** 10.1128/aem.01507-24

**Published:** 2025-01-08

**Authors:** Lauren N. Hart, Brittany N. Zepernick, Kaela E. Natwora, Katelyn M. Brown, Julia Akinyi Obuya, Davide Lomeo, Malcolm A. Barnard, Eric O. Okech, 2022-23 NSF-IRES Lake Victoria Research Consortium, Dorine Achieng, E. Anders Kiledal, Paul A. Den Uyl, Mark Olokotum, Steven W. Wilhelm, R. Michael McKay, Ken G. Drouillard, David H. Sherman, Lewis Sitoki, James Achiya, Albert Getabu, Kefa M. Otiso, George S. Bullerjahn, Gregory J. Dick

**Affiliations:** 1 Kenya Marine and Fisheries Research Institute, Kisumu, Kenya; 2 Bowling Green State University, Bowling Green, Ohio, USA; 3 University of Wisconsin—Madison, Madison, Wisconsin, USA; 4 King’s College London, London, United Kingdom; 5 Aquatic Taxonomy Specialists, Malinta, Ohio, USA; 6 African Center for Aquatic Research and Education, Ann Arbor, Michigan, USA; 7 George Mason University, Fairfax, Virginia, USA; 8 Technical University of Kenya, Nairobi, Kenya; 9 Great Lakes Institute for Environmental Research, University of Windsor, Windsor, Ontario, Canada; 10 Sigalagala National Polytechnic, Kakamega, Kenya; 11 Kisii University, Kisii, Kenya; 12 Michigan Trout Unlimited, Dewitt, Michigan, USA; 13 Technical University of Mombasa, Mombasa, Kenya; 14 Jaramogi Oginga Odinga University of Science and Technology, Bondo, Kenya; 15 University of Wisconsin—Milwaukee, Milwaukee, Wisconsin, USA; 16 Florida Gulf Coast University, Fort Myers, Florida, USA; 17 Arizona State University, Tempe, Arizona, USA; 18 Maasai Mara University, Narok, Kenya; 19 Fort LeBoeuf School District, Erie, Pennsylvania, USA; 1 Program in Chemical Biology, University of Michigan 1259 , Ann Arbor, Michigan, USA; 2 Life Sciences Institute, University of Michigan 123743 , Ann Arbor, Michigan, USA; 3 Great Lakes Center for Fresh Waters and Human Health, Bowling Green State University 1888 , Bowling Green, Ohio, USA; 4 Department of Microbiology, The University of Tennessee Knoxville 189504 , Knoxville, Tennessee, USA; 5 Large Lakes Observatory, University of Minnesota Duluth 500150 , Duluth, Minnesota, USA; 6 Biological Sciences, Bowling Green State University 110004 , Bowling Green, Ohio, USA; 7 Kenya Marine and Fisheries Research Institute 117470 , Kisumu, Kenya; 8 Department of Geography, King's College London 4616 , London, United Kingdom; 9 Department of Biology, Baylor University Department of Biology 464765 , Waco, Texas, USA; 10 Center for Reservoir and Aquatic Systems Research, Baylor University 14643 , Waco, Texas, USA; 11 Egerton University 107852 , Njoro, Kenya; 12 Department of Earth and Environmental Sciences, University of Michigan 1259 , Ann Arbor, Michigan, USA; 13 National Fisheries Resources Research Institute (NaFIRRI) 187216 , Jinja, Uganda; 14 Cooperative Institute for Great Lakes Research (CIGLR), University of Michigan 1259 , Ann Arbor, Michigan, USA; 15 Great Lakes Institute for Environmental Research, University of Windsor 177440 , Windsor, Ontario, Canada; 16 Natural Products Discovery Core, University of Michigan 1259 , Ann Arbor, Michigan, USA; 17 Department of Medicinal Chemistry, University of Michigan 1259 , Ann Arbor, Michigan, USA; 18 Technical University of Kenya 548245 , Nairobi, Kenya; 19 Kisii University 217802 , Kisii, Kenya; 20 School of Earth, Environment and Society, Bowling Green State University 1888 , Bowling Green, Ohio, USA; University of Delaware, Lewes, Delaware, USA

**Keywords:** African Great Lakes, eutrophication, *Dolichospermum*, *Microcystis*, climate change, metagenomics

## Abstract

The Winam Gulf in the Kenyan region of Lake Victoria experiences prolific, year-round cyanobacterial harmful algal blooms (cyanoHABs) which pose threats to human, livestock, and ecosystem health. To our knowledge, there is limited molecular research on the gulf’s cyanoHABs, and thus, the strategies employed for survival and proliferation by toxigenic cyanobacteria in this region remain largely unexplored. Here, we used metagenomics to analyze the Winam Gulf’s cyanobacterial composition, function, and biosynthetic potential. *Dolichospermum* was the dominant bloom-forming cyanobacterium, co-occurring with *Microcystis* at most sites. *Microcystis* and *Planktothrix* were more abundant in shallow and turbid sites. Metagenome-assembled genomes (MAGs) of *Dolichospermum* harbored nitrogen fixation genes, suggesting diazotrophy as a potential mechanism supporting the proliferation of *Dolichospermum* in the nitrogen-limited gulf. Over 300 biosynthetic gene clusters (BGCs) putatively encoding the synthesis of toxins and other secondary metabolites were identified across the gulf, even at sites where there were no visible cyanoHAB events. Almost all BGCs identified had no known synthesis product, indicating a diverse and novel biosynthetic repertoire capable of synthesizing harmful or potentially therapeutic metabolites. *Microcystis* MAGs contained *mcy* genes encoding the synthesis of hepatotoxic microcystins which are a concern for drinking water safety. These findings illustrate the spatial variation of bloom-forming cyanobacteria in the Winam Gulf and their available strategies to dominate different ecological niches. This study underscores the need for further use of genomic techniques to elucidate the dynamics and mitigate the potentially harmful effects of cyanoHABs and their associated toxins on human, environmental, and economic health.

**IMPORTANCE:**

The Winam Gulf (Kenya) is a vital resource that experiences prolific cyanobacterial harmful algal blooms (cyanoHABs). Bloom-forming cyanobacteria produce cyanotoxins, threatening human and environmental health, recreation, and fishing. However, cyanotoxin production in the gulf has not been linked to a specific type of cyanobacteria. We used DNA sequencing of whole microbial communities to track the species of cyanobacteria present across the gulf and investigate the genes responsible for synthesis of known and novel toxins. Our results reveal *Dolichospermum* as the main bloom-forming cyanobacteria in the gulf, often co-occurring with high abundance of toxigenic *Microcystis.* Over 300 unique gene clusters were found, with most predicted to encode the synthesis of uncharacterized molecules. These results provide initial insights into the diverse biosynthetic potential encoded by cyanobacteria in the Winam Gulf and underscore the need to further elucidate and investigate the effects of known and novel molecules produced in cyanoHABs in this region.

## INTRODUCTION

Cyanobacterial harmful algal blooms (cyanoHABs) are a severe, global threat to freshwater systems and are expected to intensify with climate change (1–3). The overgrowth of cyanobacteria and production of cyanotoxins threaten human and environmental health, freshwater supply, livestock, recreation, tourism, and fishing (4–6). Two of the most ubiquitous freshwater, bloom-forming cyanobacterial genera are *Microcystis*, a non-diazotroph notorious for its ability to produce toxic microcystins, and *Dolichospermum*, a filamentous diazotroph (7–10). These two genera can produce a variety of hepatotoxins, neurotoxins, and other secondary metabolites, including microcystins, saxitoxins, anatoxins, anabaenopeptins, aeruginosins, microginins, microviridins, and cyanopeptolins (10, 11).

Considerable attention has been directed toward understanding and monitoring *Microcystis-* and *Dolichospermum-*dominated cyanoHABs in North America and Europe (12–15). Yet, cyanoHAB characteristics, occurrences, and consequences in the global south remain less studied (16). Notably, Lake Victoria, the world’s second-largest lake by surface area, is experiencing rapid eutrophication and proliferation of toxic, *Microcystis-*dominated cyanoHABs (17, 18). CyanoHABs are prevalent throughout the Lake Victoria basin, which has a coastline shared by Kenya, Uganda, and Tanzania. Moreover, most cyanoHABs are concentrated within embayments such as Winam Gulf (i.e., Nyanza Gulf) (17, 18).

The Winam Gulf is a shallow inlet (average depth 7 m) near the northeastern corner of Lake Victoria that experiences year-round cyanoHABs (17). The gulf provides water for drinking, laundry, bathing, nearshore subsistence, and commercial fishing, as well as aquaculture, to Kisumu, Kenya’s third largest city, and many nearby fishing villages such as Homa Bay and Kendu Bay (directly supporting ~4.5 million people) (19, 20). A 2015 study found that 50% of households sampled near Kisumu reported sole use of raw lake water for drinking and household use, with greater than 30% of water samples from the area exceeding World Health Organization (WHO) drinking water guidelines for microcystins (21). Sixty percent of samples exceeded microcystin thresholds for children and immunocompromised individuals suggested by the United States Environmental Protection Agency, which is of particular concern due to the high prevalence of immunomodulating diseases in Kisumu like HIV and malaria (21–24). Thus, resource-dependent populations could face many routes and multifaceted effects of cyanotoxin exposure.

**Fig 1:**
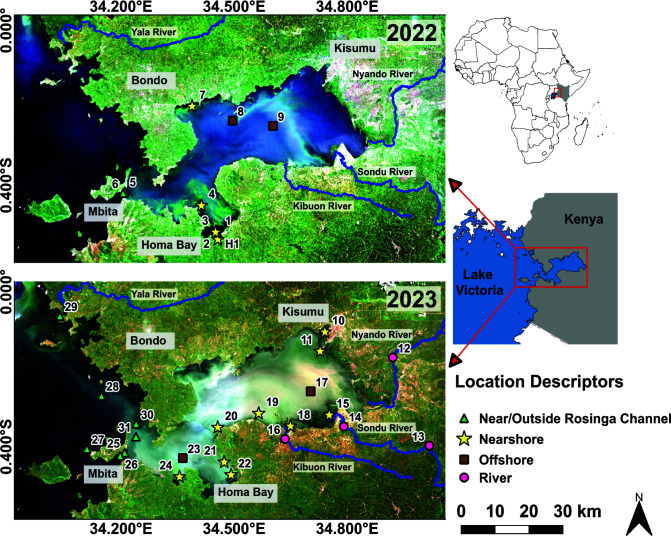
True color images of Winam Gulf, Lake Victoria, Kenya. The images were captured by Sentinel-2 on 30 June 2022 (top panel) and 26 May 2023 (bottom panel) and obtained as Level-2A (Surface Reflectance—atmospherically corrected by ESA using Sen2Cor) from Google Earth Engine. The images were collated together using QGIS LTR version 3.28.14. Sites sampled in 2022 are shown in the top panel, while sites sampled in 2023 are shown in the bottom panel. The location descriptor of a site is indicated by a symbol (star, circle, square, or triangle) with the site number indicated next to it on the map. Corresponding information for each site is reported in Table 1.

Since the 1980s, the Winam Gulf has suffered profound ecological changes due to invasive water hyacinth and other species, oxygen depletion, and increasing phytoplankton biomass (20). These changes are accompanied by substantial human population growth and increasing agricultural, industrial, urban, and wastewater contaminant runoff into the gulf (25). The main rivers flowing into the gulf are the Nyando and Sondu-Miriu Rivers which enter from the southeast corner, delivering loads of silt, nitrogen (N), and phosphorus (P) from agricultural, industrial, and wastewater sources. Smaller rivers, such as the Awach-Tende, Awach-Kibuon, Kibos, and Kisat rivers, deliver sewage and urban runoff from Kisumu (Fig. 1). Sugarcane processing, tea and cereal farming, and urban areas within the Winam Gulf catchment are the main sources of pollution, with Kisumu as a significant contributor (26, 27). Total nitrogen (TN) is higher in the gulf than the open lake, while total phosphorus (TP) is lower in the gulf than the open lake. Despite abundant sources of nitrogen delivery to the gulf, TN:TP ratios remain lower than 20, indicating nitrogen limitation relative to phosphorus and potentially selecting for diazotrophic organisms (26). Despite this, non-diazotrophic cyanobacteria are commonly found in the gulf (i.e., *Microcystis*). This indicates that there is an abundance of available nutrients in the gulf, allowing non-diazotrophs to proliferate even under nitrogen-limited conditions by employing alternative strategies (28).

**TABLE 1:** Summary of sampled stations included in the study

Station number	Site name	Sample date	Location descriptor
1	Homa Bay	24 June 2022	Nearshore
2	Homa Bay Pier	24 June 2022	Nearshore
3	Homa Bay Water Intake	24 June 2022	Nearshore
4	Soklo	24 June 2022	Nearshore
5	Mbita East^*a*^	24 June 2022	Near/Outside Rusinga Channel
6	Mbita West^*a*^	24 June 2022	Near/Outside Rusinga Channel
7	Asembo Bay	25 June 2022	Nearshore
8	Ndere Island	25 June 2022	Offshore
9	Mid Gulf	25 June 2022	Offshore
10	Kisumu Harbor	29 May 2023	Nearshore
11	Dunga	29 May 2023	Nearshore
12	Nyando River	29 May 2023	River
13	Sondu River Bridge	29 May 2023	River
14	Sondu River Shore	29 May 2023	River
15	Sondu Miriu	29 May 2023	Nearshore
16	Kibuon River	30 May 2023	River
17	Southern Mid-Gulf	29 May 2023	Offshore
18	Awach River Mouth	30 May 2023	Nearshore
19	Bala Rawi	30 May 2023	Nearshore
20	Ingra	30 May 2023	Nearshore
21	Kowuor	30 May 2023	Nearshore
22	Oluch	31 May 2023	Nearshore
23	Sikli	31 May 2023	Offshore
24	Mirunda	31 May 2023	Nearshore
25	Mbita East^*a*^	1 June 2023	Near/Outside Rusinga Channel
26	Mbita West^*a*^	1 June 2023	Near/Outside Rusinga Channel
27	Bridge Island	1 June 2023	Near/Outside Rusinga Channel
28	Bondo	2 June 2023	Near/Outside Rusinga Channel
29	Yala River Mouth	2 June 2023	Near/Outside Rusinga Channel
30	Rusinga Channel	3 June 2023	Near/Outside Rusinga Channel
31	South Rusinga	3 June 2023	Near/Outside Rusinga Channel

^*a*^ Indicates sampling at site in both years.

The climate of the Winam Gulf is characterized by two wet seasons, ranging from March to May and October to December. Two studies, from 2008 to 2009 and 2020, found maximum phytoplankton biovolumes coincide with wet (March, April, and May) and seasonal upwelling (July and August) seasons in the gulf (17, 29). Based on morphological classifications of phytoplankton, the 2008–2009 study found *Microcystis* dominates cyanoHABs in the gulf, with increased abundance of the diazotrophic cyanobacterial genus *Anabaena*, now renamed *Dolichospermum*, in September when nitrogen became more limited (30). Microcystin concentrations often exceeded WHO guidelines and increased in tandem with *Microcystis*, suggesting *Microcystis* to be the dominant microcystin producer in the gulf (17). The 2020 study identified *Microcystis* and *Dolichospermum* as the two main bloom-forming cyanobacteria at high abundance from May through July (29). Another study in 2015 and 2016 found similar phytoplankton patterns and identified *Microcystis* and *Dolichospermum* in community waters and near the drinking water intake in Kisumu (21).

Most molecular studies of the Winam Gulf have focused on fish pathogens such as *Escherichia coli*, *Vibrio cholerae*, and *Salmonella* spp. (31–33). A study by Brown et al. in 2022 used 16S rRNA gene sequencing to characterize the microbial community in the gulf, identifying the diazotrophic genus *Dolichospermum* as the dominant cyanobacterial organism (28). This study also found high copy numbers across the gulf of *cyrA* and *mcyE*, conserved genes in the biosynthetic gene clusters (BGCs) for cylindrospermopsin and microcystin, respectively (28). Despite these investigations, the producer(s) of these cyanotoxins in the gulf remains unknown. Additionally, the functional and biosynthetic capabilities of bloom-forming cyanobacteria that contribute to their fitness across different regions of the gulf have not been studied.

The aim of this study was to genetically characterize the functional and biosynthetic potential of bloom-forming cyanobacteria in the Winam Gulf using whole-genome shotgun metagenomic sequencing. We conducted a 2-year sampling expedition (2022–2023) from coastal, offshore, and river sites within and outside of the gulf. We found that cyanoHAB events were not limited to coastal zones, or even within the gulf, and were most prolific near Homa Bay in 2022 and outside of the gulf near Bondo and the Yala River mouth in 2023. These cyanoHAB events were dominated by *Dolichospermum*, with *Microcystis* co-occurring but at lower abundance. Highly diverse and abundant BGCs were encoded by many genera of cyanobacteria, with the most biosynthetic potential encoded at sites with the highest abundance of bloom-forming cyanobacteria. Most BGCs could not be linked to a known biosynthesis product, highlighting the novel chemical repertoire encoded by cyanobacteria in the gulf. Lastly, our data suggest that *Microcystis* is the main and sole producer of microcystin in the gulf during the sampling period.

## RESULTS

### Water physicochemistry indicates nitrogen limitation throughout the gulf

**Fig 2:**
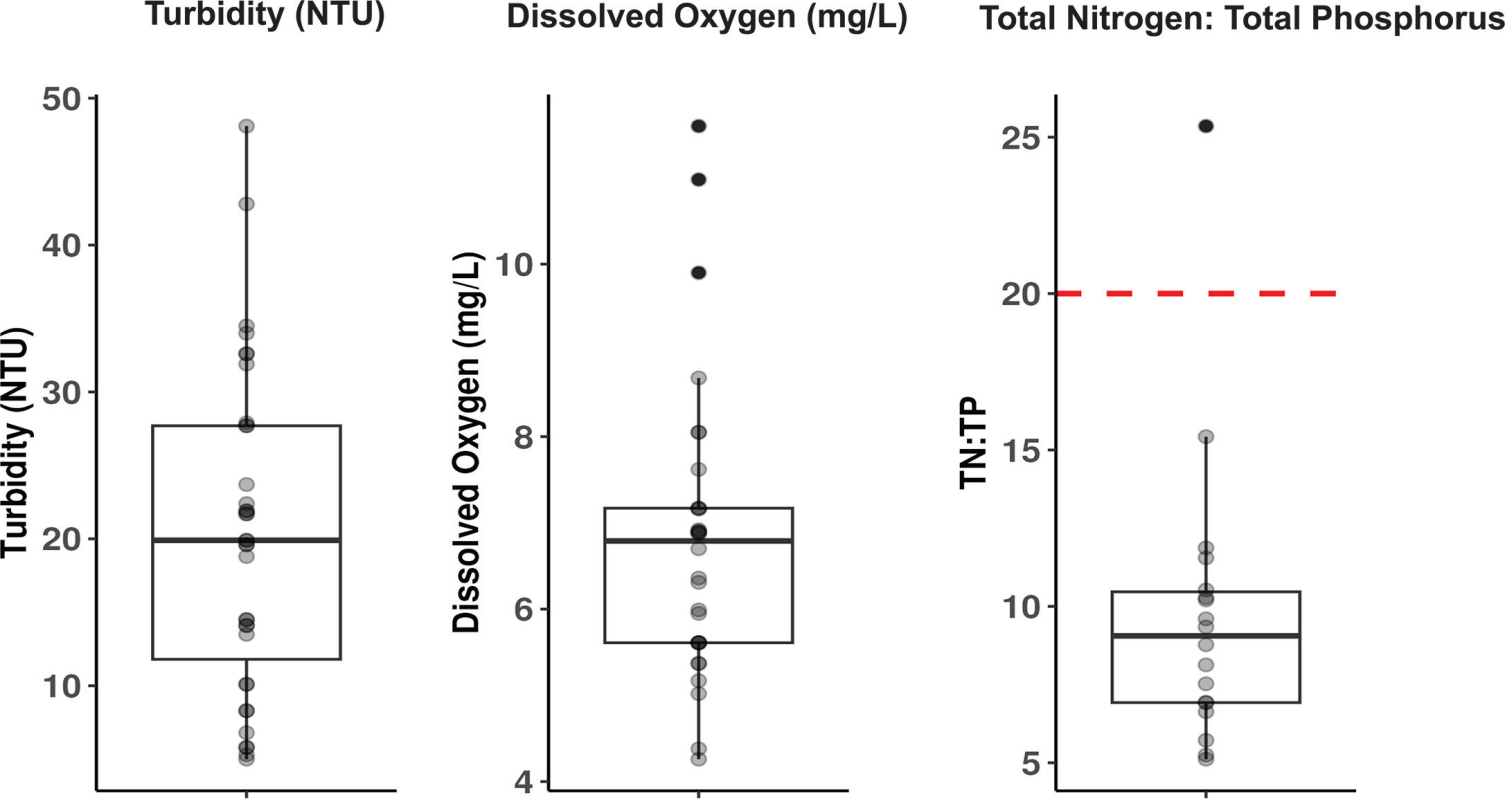
Turbidity (NTU), dissolved oxygen (mg/L), and TN:TP (molar) ratio measurements. Individual data points for each variable are plotted on top of each box plot. The red line in the TN:TP panel indicates the threshold for nitrogen-limitation, with all values below it indicating nitrogen-limiting scenarios according to Guildford and Hecky (2000) (34). TN:TP was only measured for sites in 2023.

Thirty-one sites throughout the gulf and nearby rivers were sampled for metagenomic analysis in May and June of 2022 and 2023 (Fig. 1; Table 1). The sites sampled span spatial, temporal, physical, and chemical (physicochemical) gradients. Physicochemical analyses were performed at each site to infer water quality (Fig. 2; Table S1), excluding river sites. Turbidity varied across sites ranging between 10 and 48.1 nephelometric turbidity units (NTUs), with a median of 23.7 NTUs. Dissolved oxygen (DO) measured between 4.26 and 11.6 mg/L across sites, with sites within the Rusinga Channel (26, 27, and 31), on the higher end of the range measured at 9.9, 10.98, and 11.6 mg/L, respectively. TN and TP were measured in 2023, and ratios (TN:TP) ranged between 5.1 and 25 with a median of 9.06. All sites except site 19 fell below a TN:TP ratio of 20, suggestive of nitrogen limitation (34) (Fig. 2). Site 19 on the southern coast of the gulf had a TN:TP ratio of 25, falling in the ambiguous nitrogen or phosphorus limited range.

### River and gulf sites vary in microbial community makeup

**Fig 3:**
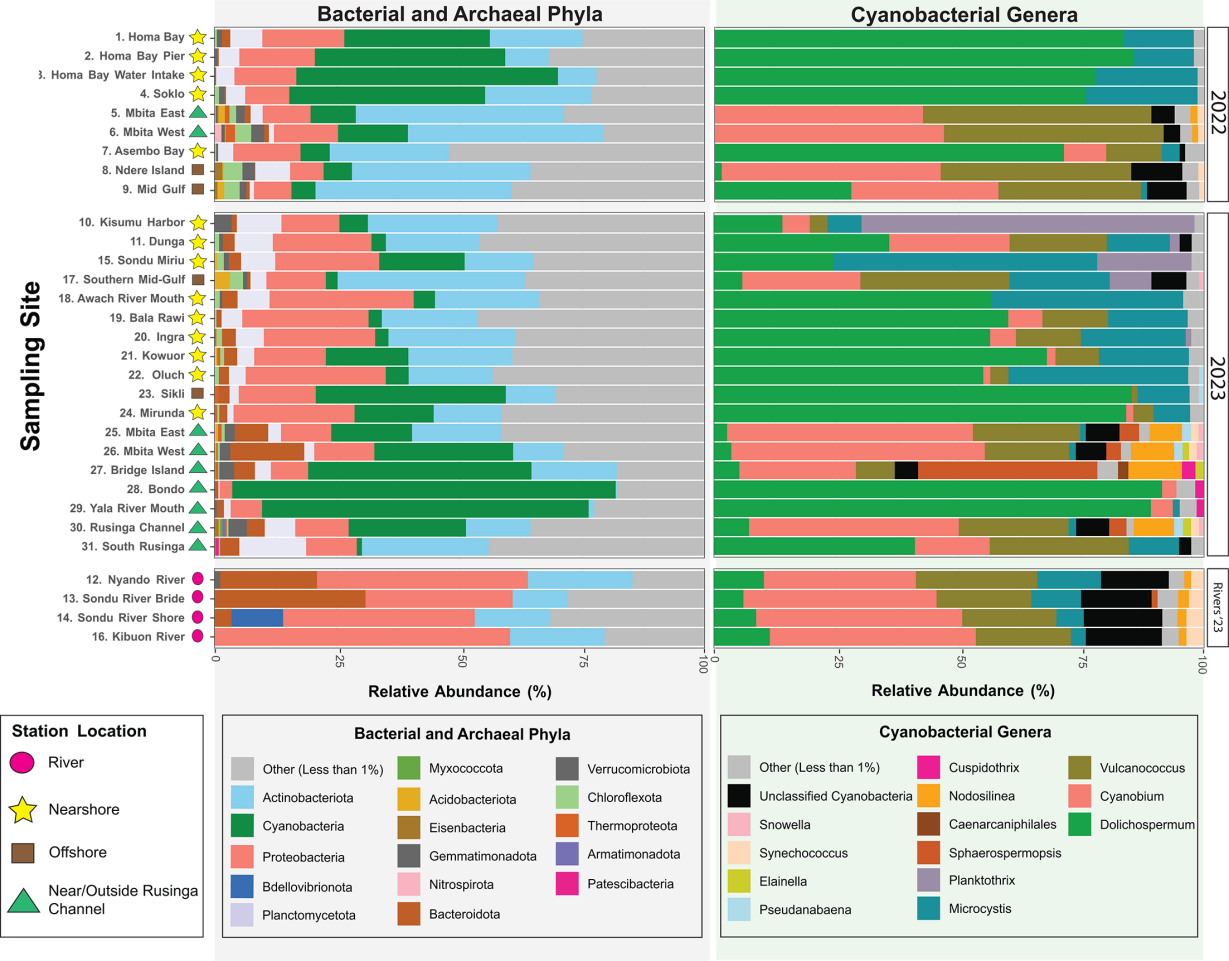
Microbial and archaeal composition makeup in Winam Gulf, LV. Relative abundance (percentage) of bacterial and archaeal phyla (gray panel) and cyanobacterial genera (green panel) from sampled sites in 2022 and 2023 is shown. The phyla and genera representing less than 1% of the population were grouped in the “Other” category. Cyanobacteria with no taxonomic assignment for genus were labeled as “unclassified cyanobacteria.” Unmapped reads were excluded from this analysis, and mapped reads were normalized to 100% for phyla and genera. Station location indicators are shown using symbols after the name of the site number and name on the y-axis.

Overall, the microbial community composition of the gulf closely resembled other eutrophic lakes with the phyla *Actinobacteria*, *Cyanobacteria*, *Proteobacteria*, and *Planctomycetota* being highly abundant (35) (Fig. 3; Table S2). The communities in 2022 and 2023 were similar in their compositions and dominant microbial phyla. The community composition at river sites (sites 12, 13, 14, and 16) diverged from that of gulf sites primarily because of lower *Cyanobacteria* (*P* = 0.1) and significantly higher *Bacteroidota* (*P* < 0.001) abundance observed in the samples. Cyanobacteria represented less than 1% of the total microbial community in each river site. Cyanobacteria were present at all sites, with relative abundance ranging between approximately 1% at site 31 and 78% at site 28.

**Fig 4:**
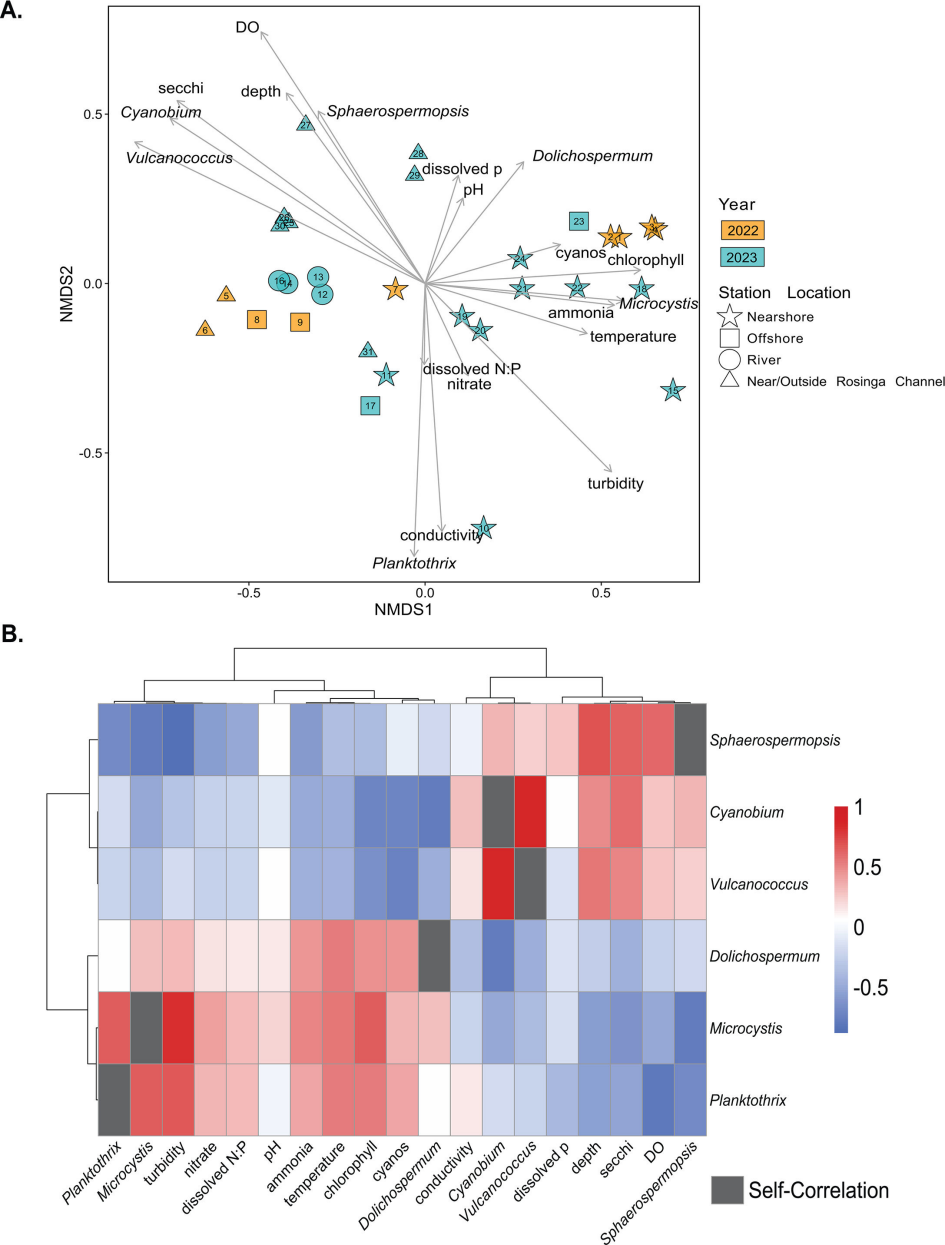
Cyanobacterial composition of Winam Gulf in relation to physicochemical parameters. (**A**) Non-metric multidimensional scaling (NMDS) analysis of the relative abundance of all cyanobacterial genera from each site, in relation to all other microbial community members (stress = 0.074). Labeled arrows show the direction and strength of the relationship between the ordination axes and associated environmental parameters and the relative abundance of the top six most abundant cyanobacterial genera across the sites. Shapes of ordination points indicate station location. Samples from 2022 are colored in amber and samples from 2023 in teal. (**B**) Spearman correlation coefficients between the six most abundant cyanobacterial genera across the sample sites and environmental parameters. Rows and columns are hierarchically clustered using correlation. Gray squares indicate self-correlation.

### *Dolichospermum* dominates large CyanoHAB events detected in distinct gulf regions

*Dolichospermum*, *Cyanobium*, *Vulcanococcus*, and *Microcystis* were the major cyanobacterial genera found in the gulf and rivers (Fig. 3; Table S2). Minor cyanobacterial genera present across the sites were *Snowella*, *Synechococcus*, *Elainella*, *Pseudanabaena*, *Cuspidothrix*, *Nodosilinea*, and *Caenarcaniphilales. Sphaerospermopsis* and *Planktothrix* had high relative abundance at sites 27 and 10, respectively, making up more than 36% and 65% of the cyanobacterial community at these two sites, respectively. S*phaerospermopsis* and *Planktothrix* were found at four other sites at an abundance below 5% and 20% of the total cyanobacterial community, respectively. Cyanobacteria unclassified at the genus level were a substantial portion of the community (1%–16%) at most sites.

River sites had similar cyanobacterial compositions despite their lower cyanobacterial abundance. *Cyanobium* and *Vulcanococcus* represented greater than 50% of the cyanobacterial population in river sites, with *Dolichospermum* and *Microcystis* representing less than 15% individually.

Across the Gulf, there were two major scenarios, with cyanobacterial community composition dominated by either bloom-forming cyanobacterial genera (*Dolichospermum* and *Microcystis*; sites 1, 2, 3, 4, 7, and 18–24) or picocyanobacterial genera (*Cyanobium* and *Vulcanococcus*; sites 5, 6, 8, 9, 25, 26, 30, and 31). In 2022, there was a large cyanoHAB event made up of *Dolichospermum* and *Microcystis* near Homa Bay, together making up approximately 98% of the cyanobacterial community. In 2023, *Dolichospermum* and *Microcystis* dominated sites along the southern and southwestern coast of the gulf, beginning near the Awach River mouth through Homa Bay and toward Mbita, together making up between 77% and 95% of the cyanobacterial community at these sites. At the sites where *Dolichospermum* and *Microcystis* co-occurred, *Dolichospermum* was more abundant than *Microcystis* by approximately 25%–60%. In a few instances, a single genus of cyanobacteria dominated the cyanobacterial assemblage: *Planktothrix* at site 10 in Kisumu harbor; *Microcystis* at site 15 near the Sondu River mouth; *Sphaerospermopsis* at site 27 off Rusinga Island in the open waters of Lake Victoria; and *Dolichospermum* at sites 28 and 29 outside the gulf near Bondo and the Yala River mouth.

### Relationships between dominant cyanobacterial genera and environmental conditions

Cyanobacterial composition was assessed in relation to selected environmental parameters through non-metric MultiDimensional Scaling (NMDS; Fig. 4A). River sites and sites dominated by picocyanobacteria *Cyanobium* and *Vulcanococcus* clustered together and showed a positive relationship with DO. Sites dominated by *Dolichospermum* and *Sphaerospermopsis* clustered near each other and had a positive relationship with dissolved phosphorus, pH, and depth. Sites with mixed communities of bloom-forming cyanobacteria including *Microcystis* and *Planktothrix* formed a large cluster and had a positive relationship with ammonia, nitrate, and turbidity.

The relative abundance of dominant cyanobacterial genera (*Dolichospermum*, *Cyanobium*, *Vulcanococcus*, *Microcystis*, *Planktothrix*, and *Sphaerospermopsis*) was assessed for their relationship with environmental parameters using Spearman’s correlation analysis (Fig. 4B; Table S3). *Dolichospermum*, *Microcystis*, and *Planktothrix* shared similar relationships with environmental parameters. These genera had strong positive correlations (greater than or equal to 0.5) with ammonia, temperature, chlorophyll, and turbidity (*P* < 0.001). This group was strongly negatively correlated (less than −0.5) with depth and DO (*P* < 0.001). *Sphaerospermopsis*, *Cyanobium*, and *Vulcanococcus* clustered together based on their relationships with environmental parameters. *Sphaerospermopsis* was strongly positively correlated with DO, depth, and dissolved phosphorus, while strongly negatively correlated with ammonia, dissolved N:P, nitrate, and turbidity (*P* < 0.001). *Sphaerospermopsis’* negative correlation with dissolved N:P levels suggests it to be potentially more fit in nitrogen-limiting waters, where this negative correlation most often occurs at dissolved N:P ratios less than 1 (indicative of nitrogen deficiency). *Cyanobium* and *Vulcanococcus* were strongly positively correlated with depth and strongly negatively correlated with ammonia, temperature, and cyanobacterial detection (“cyanos”) via the use of an AlgaeTorch (bbe moldaenke GmnH, Schwentinental, Germany; *P* < 0.001).

### Phylogenomic analysis reveals low intra-genus genetic diversity and novel clades of bloom-forming cyanobacteria from the gulf

**Fig 5:**
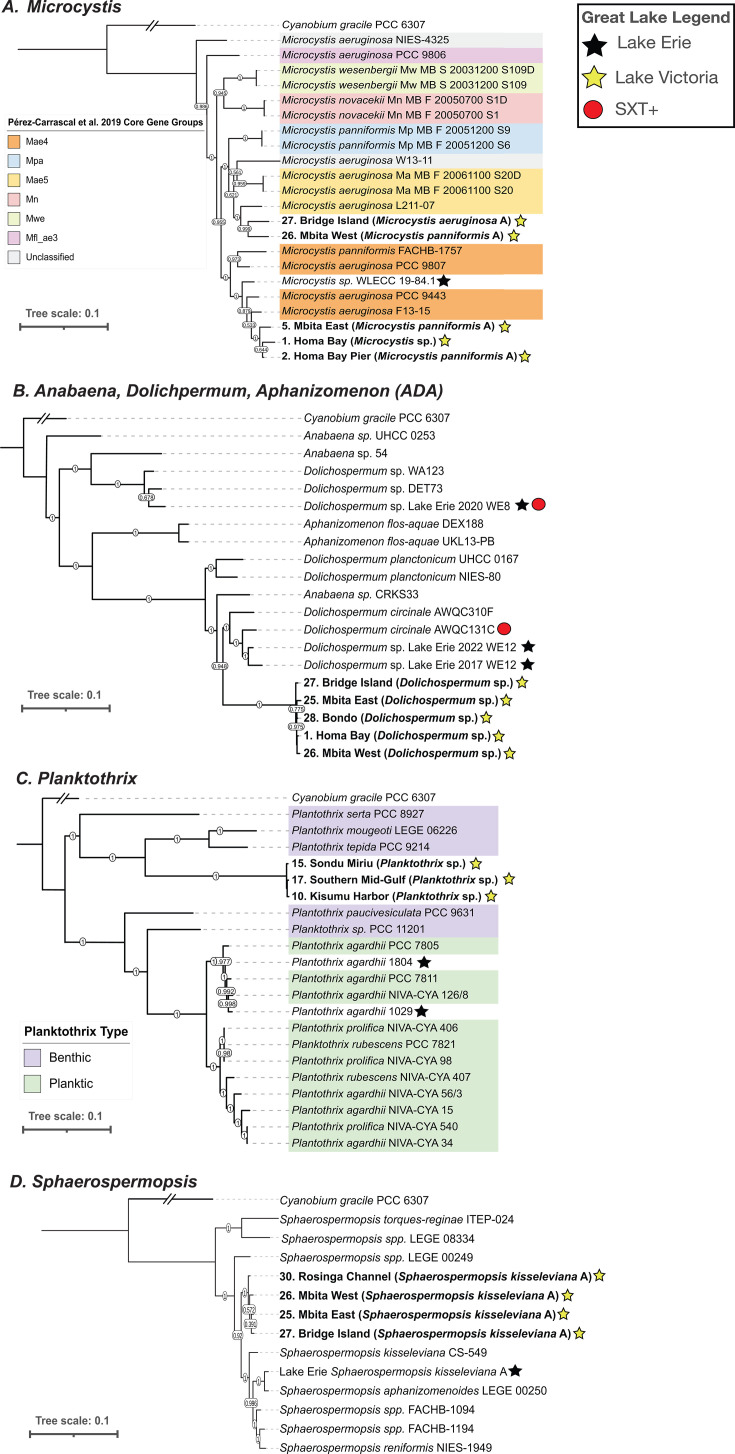
Phylogenomic analysis of dominant bloom-forming cyanobacteria from sampled sites with taxonomic annotations from Genome Taxonomy Database (GTDB) (40). (**A**) *Microcystis* phylogenomic tree built with five representative MAGs, reference genomes, and *Cyanobium gracile* PCC 6307 as the outgroup. Colored ranges indicate clades as outlined in reference (36). (**B**) *Dolichospermum* phylogenomic tree built with five representative MAGs, reference genomes, and *Cyanobium gracile* PCC 6307 as the outgroup. The presence of a red circle is indicative of the presence of the saxitoxin gene cluster within the genome (38). (**C**) *Planktothrix* phylogenomic tree built with three representative MAGs, reference genomes, and *Cyanobium gracile* PCC 6307 as the outgroup. Benthic or planktic designations for reference genomes are indicated as colored ranges (41). (**D**) *Sphaerospermopsis* phylogenomic tree built with four representative MAGs, reference genomes, and *Cyanobium gracile* PCC 6307 as the outgroup.

Assembly and binning of metagenomic sequence reads produced 203 high-quality cyanobacterial metagenome-assembled genomes (MAGs; ≥90% completion and ≤10% contamination; Table S4). Dereplication at 99.5% average nucleotide identity produced a total of 17 representative MAGs of the genera *Microcystis* (five MAGs), *Dolichospermum* (five MAGs), *Planktothrix* (three MAGs), and *Sphaerospermopsis* (four MAGs; Table 2). Phylogenomic analysis showed that *Microcystis* MAGs from the gulf formed two distinct clades (Fig. 5A). Three MAGs from sites 1, 2, and 5 clustered with the Mae4 clade (36), including strain LE19-84.1 isolated from Western Lake Erie (37). Two MAGs from sites 26 and 27 fell within the Mae5 clade. *Dolichospermum* MAGs from sites 1, 25, 26, 27, and 28 formed a single, tight sister clade to a clade of *Dolichospermum circinale* and two MAGs from Lake Erie, including one MAG that has the saxitoxin BGC (Fig. 5B) (38). *Planktothrix* MAGs from sites 10, 15, and 17 formed a single clade most closely related to *Planktothrix* strains with benthic properties (Fig. 5C). *Sphaerospermopsis* MAGs from sites 25, 26, 27, and 30 formed a single, tight clade most closely related to *Sphaerospermopsis* sp. LEGE 00249, an isolate from a reservoir in Portugal (Fig. 5D) (39).

**TABLE 2:** Summary and statistics of high-quality, dereplicated cyanobacterial MAGs

MAG	Site	Completeness (%)	Contamination (%)	Strain heterogeneity (%)	Species (based on GTDBtk annotations)	Size (Mbp)	No. of contigs	GC content (%)	N50	NCBI accession
samp_4315_VAMB_280	Mbita East	92.91	5.74	60	*Microcystis panniformis* A	4.230761	2,568	43.5	2,116	SAMN41711324
samp_4322_concoct_168	Homa Bay Pier	98.79	1.24	55.56	*Microcystis panniformis A*	5.518758	724	42.6	11,220	SAMN41711381
samp_4325_VAMB_332	H1. Homa Bay Coast	95.24	6.77	37.21	*Microcystis* sp.	5.43631	4,522	42.8	1,77	SAMN41711408
samp_4444_concoct_1899	Mbita West	90.3	7.15	69.05	*Microcystis panniformis* A	3.847615	1,713	43.2	2,584	SAMN41711474
samp_4445_concoct_1450	Bridge Island	90.56	6.79	78.05	*Microcystis aeruginosa* H	3.729636	1,804	43.1	2,269	SAMN41711483
samp_4326_maxbin_35	H1. Homa Bay Coast	97.22	0.22	0	*Dolichospermum* sp.	4.077973	370	37.5	16,022	SAMN41711415
samp_4443_metadecoder_676	Mbita East	93.74	1.01	33.33	*Dolichospermum* sp.	3.767753	443	37.2	11,273	SAMN41711470
samp_4444_maxbin_761	Mbita West	91.61	0.81	33.33	*Dolichospermum* sp.	3.834118	345	37.1	15,823	SAMN41711477
samp_4445_metadecoder_820	Bridge Island	95.11	0.44	50	*Dolichospermum* sp.	3.814968	232	37.1	24,166	SAMN41711487
samp_4446_metabat2_471	Bondo	90.07	9.23	58.33	*Dolichospermum* sp.	4.539608	629	36.9	9,935	SAMN41711489
samp_4432_maxbin_231	Kisumu Harbor	97.82	0.95	80	*Planktothrix* sp.	5.017227	441	39.3	19,782	SAMN41711420
samp_4434_metabat2_31	Sondu Miriu	98.69	1.24	100	*Planktothrix* sp.	5.003513	421	39.2	18,186	SAMN41711432
samp_4435_metabat2_87	Southern Mid-Gulf	98.47	1.69	63.64	*Planktothrix* sp.	5.119342	423	39.2	18,944	SAMN41711440
samp_4443_maxbin_260	Mbita East	91.67	0.44	33.33	*Sphaerospermopsis kisseleviana* A	4.743136	543	37.6	11,832	SAMN41711466
samp_4444_maxbin_786	Mbita West	92.89	1.12	83.33	*Sphaerospermopsis kisseleviana* A	4.915663	624	37.6	10,851	SAMN41711478
samp_4445_VAMB_547	Bridge Island	89.96	7.53	77.55	*Sphaerospermopsis kisseleviana* A	6.110041	1,452	37.5	6,049	SAMN45857663
samp_4448_concoct_1871	Rusinga Channel	93.7	1.78	77.78	*Sphaerospermopsis kisseleviana* A	5.020957	595	37.7	10,901	SAMN41711493

### Nitrogen fixation genes harbored primarily by *Dolichospermum* and identified at sites with large bloom events

**Fig 6:**
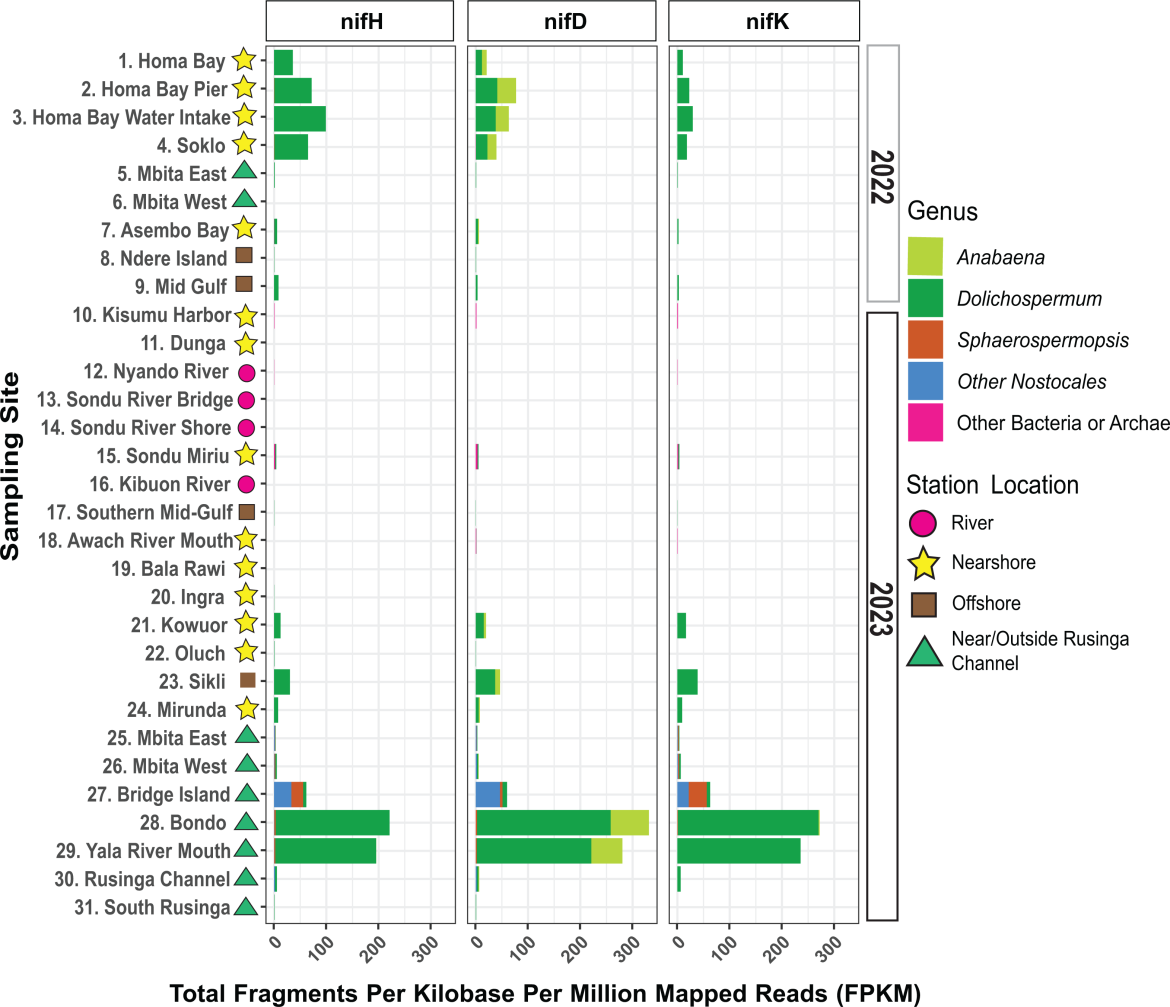
Relative abundance of *nifH*, *nifD*, and *nifK* genes across sites. Reads were aligned to *nifH*, *nifD*, and *nifK* genes from NFixDB and normalized using the total fragments per kilobase per million mapped reads (FPKM) metric (Equation 1) (43). The y-axis shows site names with their location descriptor symbol. The x-axis shows the FPKM of each *nif* gene in each sample. Bars are colored based on the genus the *nif* gene was extracted from.

Due to nitrogen limitation in the gulf and its potential selection for diazotrophic organisms, we assessed the microbial community’s ability to fix nitrogen by identifying *nif*HDK genes (42). *Dolichospermum* and *Anabaena*, two members of the *Anabaena/Dolichospermum/Aphanizomenon* (ADA) clade, had the most *nif*HDK gene matches, and very few hits were to bacteria outside of the Cyanobacteria phylum (Fig. 6) (30). *nif*HDK genes belonging to ADA clade members were found at sites where there were large cyanoHAB events in 2022 and 2023. The only instance where the three ADA clade members did not harbor most of the *nif* genes was at site 27 near Bridge Island where *Spaherospermopsis*, a closely related genera, dominated the community (Fig. 3) (10). At this site, *Sphaerospermopsis* and other *Nostacales* cyanobacteria were responsible for the *nif*HDK genes detected.

### Cyanotoxin- and cyanopeptide-encoding BGCs detected in regions with and without visible cyanoHABs

**Fig 7:**
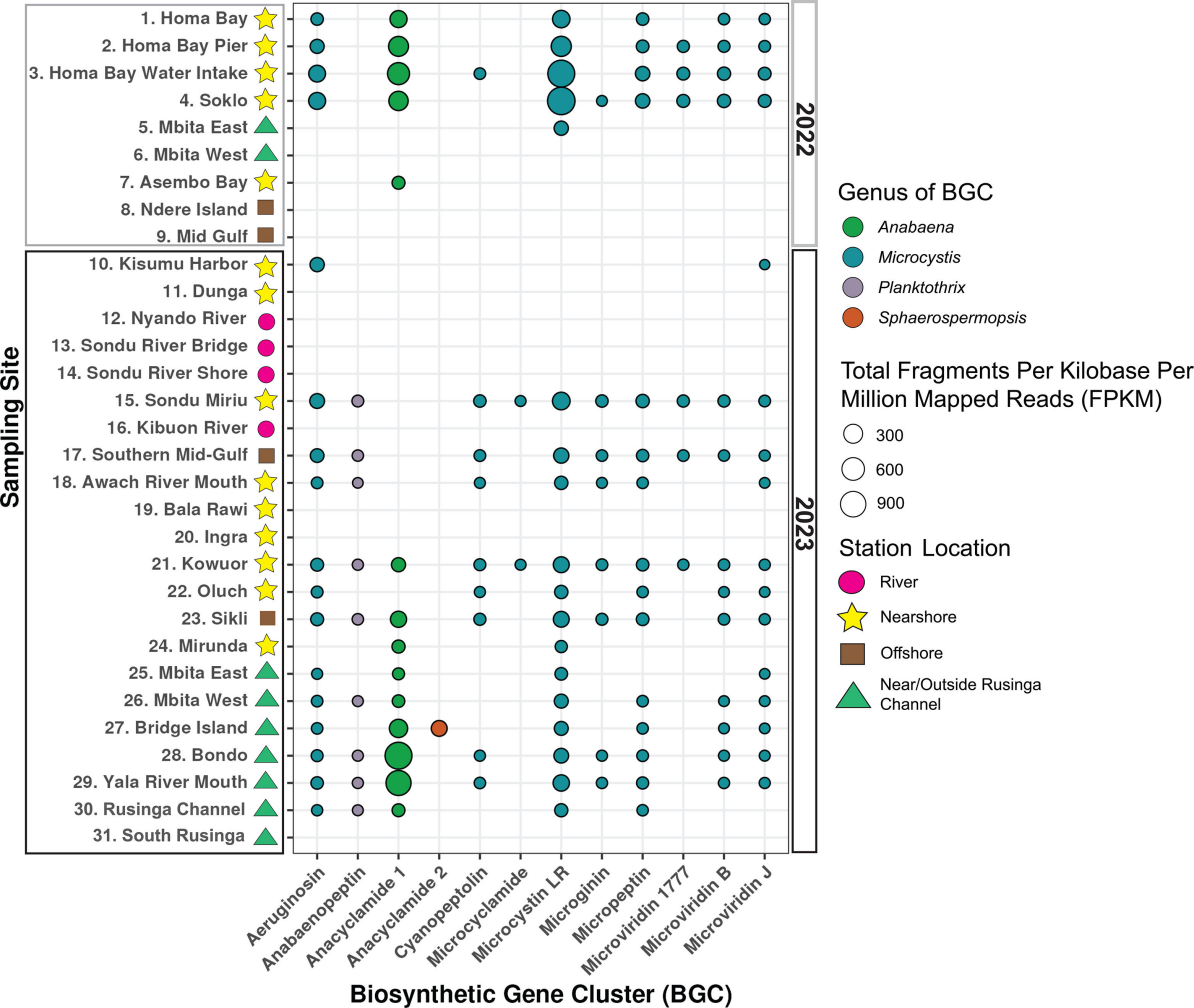
Known cyanopeptide BGC relative abundance across sites. Station numbers, names, and their location indicator symbols are shown on the y-axis, with BGCs encoding the biosynthesis of cyanopeptides from MIBiG (accessed December 2023) shown on the x-axis (44). The FPKM (total fragments per kilobase per million mapped reads) of each whole BGC is shown as bubble size (Equation 2). Bubbles are colored based on the cyanobacterial genus from which the reference BGC originates.

Mapping metagenomic sequence reads to BGCs from the MiBIG database (44) revealed high spatiotemporal variation in genetic potential for biosynthesis of cyanotoxins and cyanopeptides (Fig. 7). No cyanobacterial BGCs were found in the four river sites, or at sites 6, 8, 9, 11, 19, 20, and 31, which had limited abundance of cyanobacteria. BGCs encoding metabolites from the classes aeruginosins, anacyclamide, microcystins, micropeptins, and microviridins were most common, detected in over half of the sites. The microcystin and *Anabaena* anacyclamide 1 BGCs were the most abundant BGCs across the data set. Although genes encoding cylindrospermopsin (28, 45) did not assemble using our metagenomic approach, read mapping revealed the presence of four highly conserved cylindrospermopsin genes (*cyr*AEBC) at a low abundance at site 27 near Bridge Island (Fig. S1). Deeper sequencing could fully elucidate this BGC and its taxonomic identity in the gulf.

### Genus-specific biosynthetic potential revealed through over 300 cryptic BGCs

**Fig 8:**
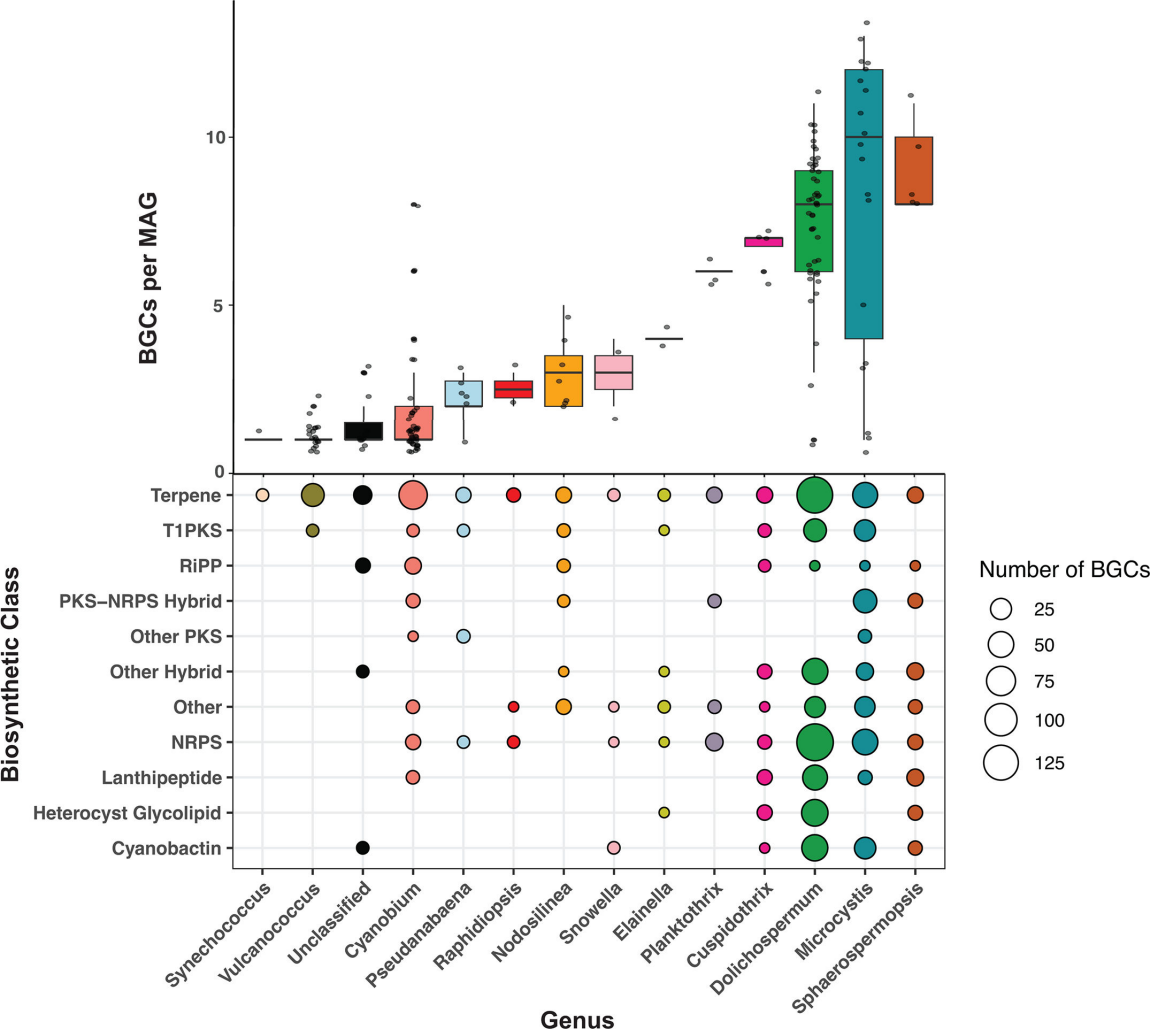
BGC richness and diversity in dominant cyanobacterial genera from the gulf. The top panel shows the average number of BGCs per MAG from 14 different cyanobacterial genera. The bottom panel shows the number of BGCs (bubble size) from each biosynthetic class (y-axis) identified from each cyanobacterial genus (x-axis) by antiSMASH v7.0 on the x-axis (46). Genus names are not italicized for readability.

BGCs were mined from cyanobacterial MAGs to assess total biosynthetic potential of the Winam Gulf cyanobacterial assemblage. One thousand BGCs from cyanobacterial MAGs were identified, 300 of which were unique, from 11 different biosynthetic classes and 14 different cyanobacterial genera (Fig. 8). Notably, all but three of these BGCs did not match any known BGCs in the MiBIG database, indicating their cryptic nature (Fig. S2). BGC richness differed between the 14 cyanobacterial genera. *Synechococcus*, *Vulcanococcus*, *Cyanobium*, and the unclassified cyanobacterial genera groups all encoded an average of one BGC per MAG, with *Cyanobium* encoding the largest range of BGCs per MAG, ranging between 1 and 8. *Pseudanabaena*, *Raphidiopsis*, *Nodosilinea*, *Snowella*, and *Elainella* encoded an average of two to four BGCs per MAG. *Planktothrix*, *Cuspidothrix*, *Dolichospermum*, *Microcystis*, and *Sphaerospermopsis* encoded the most BGCs, ranging between 6 and 10 BGCs per MAG on average.

Terpenes, the largest and most structurally diverse class of natural products including compounds such as geosmin, were encoded by all 14 cyanobacterial genera analyzed (47). The next most common biosynthetic class was non-ribosomal peptide synthetases (NRPSs), a pathway known for its synthesis of cyanopeptides like anabaenopeptins and aeruginosins (48, 49). Cyanobactin BGCs encoded by *Microcystis* and *Dolichospermum* formed gene cluster families (GCFs) with known anacyclamide BGCs from the MiBIG database (Fig. S2). BGCs putatively encoding microviridin from the ribosomally synthesized and post-translationally modified peptide (RiPP) biosynthetic class were encoded by *Planktothrix*, *Microcystis*, and *Sphaerospermopsis* (Fig. S2). *Microcystis* was the only cyanobacterial genus with MAGs encoding microcystins (Fig. S2 and S3). BGCs that could not be grouped into these larger biosynthetic classes were categorized as “Other” and were putatively annotated as RiPP recognition element (RRE)-containing, ectoine, lassopeptide, spliceotide, arylprolene, crocagin, and mycosporine-like.

### Biosynthetic potential converges with site-specific cyanobacterial composition

**Fig 9:**
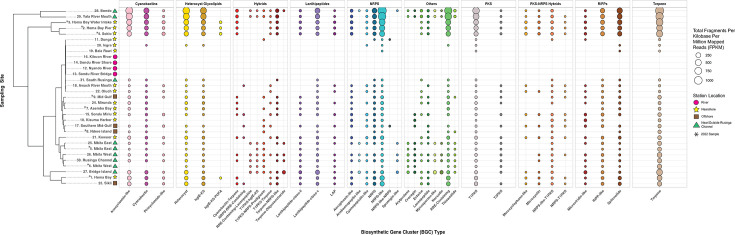
Total BGC relative abundance across the Winam Gulf. Station numbers, names, and their location indicator symbols are shown on the y-axis, with BGC groups shown on the x-axis. BGCs are grouped into broader biosynthetic or product classifications, labeled as the top facet strips (Table S3). The total fragments per kilobase per million mapped reads (FPKM) of each BGC was calculated (Equation 2), and the size of a point corresponds to the total FPKM of all BGCs of a particular BGC type indicated on the x-axis. Bubbles are colored to differentiate BGC groups. Samples on the y-axis are hierarchically clustered using the complete-linkage method.

The relative abundance of cyanobacterial BGCs varied across the gulf (Fig. 9). BGC composition at each site converged with cyanobacterial composition, particularly with the dominating cyanobacterial genera at each site (Fig. S4). Specifically, sites with a high abundance of bloom-forming cyanobacteria (1–4, 23, and 27–29) had increased abundances of most BGC classes and clustered together based on the site’s biosynthetic potential. BGCs putatively encoding pathways related to Anacyclamide-like, cyanobactin, heterocyst glycolipids, Lanthipeptide class V, NRPS, NRPS-like, RRE-containing, type 1 polyketide synthase, RiPP-like, spliceotide, terpene biosynthetic classes, and pathways were the most common and abundant BGC types across sites.

## DISCUSSION

The Winam Gulf of Lake Victoria experiences prolific, year-round cyanoHABs, previously shown to be dominated by *Microcystis* with periodic occurrences of *Dolichospermum* based on morphological classifications (17, 50). However, due to limited molecular research on these blooms, little is known about the bloom-forming cyanobacterial function and biosynthetic potential supporting year-round growth in this region. In this study, we employed metagenomics to establish a molecular snapshot of the microbial communities found along spatial and physicochemical gradients in the Winam Gulf between 2 consecutive years. Subsequently, we investigated the functional and biosynthetic potential encoded by bloom-forming cyanobacteria in the gulf to determine potential strategies used by these organisms to grow and produce toxic metabolites.

### Nitrogen limitation may provide a competitive advantage for diazotrophs during cyanoHABs

Two, large cyanoHAB events dominated mainly by *Dolichospermum* (56%–91% of the cyanobacterial community) and *Microcystis* (14%–39% of the cyanobacterial community) were detected near Homa Bay in 2022 and outside the gulf near Bondo and the Yala River mouth in 2023 (Fig. 1). Toxic cyanoHAB events, due to microcystins, in Homa Bay have presented exposure risks to this fishing community of ~60,000 residents (21, 28, 51). CyanoHABs have also been previously reported in the Yala River mouth region, which has excessive turbidity, declining water quality, and is a large source of anthropogenic pollution to Lake Victoria (52, 53). While this region is not as regularly monitored for cyanoHABs and cyanotoxins due to its location outside the gulf, our results provide further evidence that it should be considered in future monitoring schemes.

Organisms from the ADA clade and *Sphaerospermopsis* harbored essential genes for nitrogen fixation, such as the nitrogenase operon (*nif*HDK) and heterocyst glycolipid synthesis gene clusters, where cyanoHABs were detected in 2022 and 2023 (54). To our knowledge, this is the first report of the genus *Sphaerospermopsis*, a potentially toxigenic and diazotrophic cyanobacterium, in the Winam Gulf. These results suggest that diazotrophic cyanobacterial genera might be employing nitrogen fixation as a competitive advantage over non-diazotrophic competitors that are reliant on fixed forms of nitrogen. Low TN:TP ratios at cyanoHAB sites in 2023 (34) and 2005 (26) also indicate nitrogen limitation, with less severe nitrogen limitation closer to and outside the Rusinga channel (34). Although TN:TP was not measured in 2022, dissolved nitrate:soluble reactive phosphorus (DN:SRP) ratios were all below 1, suggestive of nitrogen deficiency (17). These results concur with Brown et al., which identified *Dolichospermum* as a dominant bloom-forming cyanobacteria in the gulf in 2022 but differ from previous reports that identified *Microcystis* as the dominating bloom-forming cyanobacteria in most seasons since 2008 in this region (17, 28, 55). Investigations of the phytoplankton community of the gulf in 2020 also identified *Microcystis* at slightly higher abundance than *Dolichospermum* in May and June (29). Data collection on an annual basis is vital to elucidate how changing landscapes and populations in this region are influencing cyanoHAB community structures with different toxin threats that require variant monitoring and management approaches.

According to additional studies on subtropical water bodies experiencing cyanoHABs made up of both *Microcystis* and diazotrophic cyanobacteria, high *nif*HDK (nitrogenase) gene expression was observed after large *Microcystis* blooms, with diazotrophs potentially acting as “replenishers” of bioavailable nitrogen (56). Gene expression data are needed to identify if diazotrophic cyanobacteria in the Winam Gulf serve this role in the ecosystem and could help explain the gulf’s seasonal bloom succession patterns.

### Non-diazotrophic cyanobacteria were identified at lower abundance in gulf regions with distinct physicochemical conditions

*Microcystis* and *Planktothrix*, non-diazotrophs, were at lower relative abundance at sites with large cyanoHAB events (between 1% and 39% of the cyanobacterial community). However, *Microcystis* was observed at higher relative abundance at sites in 2023 with higher turbidity on average (between 36% and 59% of the cyanobacterial community). Although these sites were nitrogen limited, the high turbidity at these sites may give *Microcystis* an advantage in comparison to deeper and less turbid regions of the gulf. This is due to *Microcystis*’ ability to form gas vesicles and thus reach the surface to circumvent light limitation, plausibly caused by the shallow depth and well-mixed water column at these sites, therefore generating shading for their non-buoyant competitors (57). *Planktothrix*, which was found to have close genomic similarity to previously characterized benthic *Planktothrix* isolates, was the dominant cyanobacterial genera in Kisumu Harbor, the shallowest site in the study (5.4 m), at the time of sampling (up to 67% of the cyanobacterial community). No *Planktothrix nif* genes were identified, indicating that *Planktothrix* was likely using strategies other than diazotrophy to overcome nitrogen limitation and proliferate in this region of the gulf (41, 58).

At sites where cyanobacteria were detected at lower abundance compared to other microbial phyla, *Cyanobium* and *Vulcanococcus* (picocyanobacteria) were the most abundant cyanobacterial genera, each making up almost half of the cyanobacterial community at these sites. There is little documentation of the picocyanobacterial community in the Winam Gulf likely due to their small cell size and thus difficult morphological classification (59). *Cyanobium* is cosmopolitan, found across the globe, and is well known for its ability to scavenge nitrogen and use light efficiently, potentially leading to its success in this ecosystem, while *Vulcanococcus* is not as well documented (60, 61). While picocyanobacteria typically do not have many BGCs, MAGs recovered in this study do encode some BGCs from terpene, PKS, and NRPS biosynthetic pathways, with *Cyanobium* encoding a far more diverse set of BGCs than *Vulcanococcus*. This finding underscores the need to include picocyanobacteria in future investigations of cyanoHABs in the Winam Gulf and freshwater systems broadly as it relates to bloom dynamics and human health (62).

### Low intra-genus diversity among bloom-forming cyanobacteria in the gulf compared to temperate systems

The main bloom-forming cyanobacteria (*Dolichospermum*, *Microcystis*, *Sphaerospermopsis*, and *Planktothrix*) showed low intra-genus diversity across the sites, recovering only 3–5 MAGs from each genus. For *Microcystis* and *Planktothrix*, this result contrasts with findings from Lake Erie, where isolates from these genera share much lower average nucleotide identities (37, 63). Conversely, the results for *Microcystis* align with findings from subtropical lakes like Lake Okeechobee (Florida, USA) and Lake Taihu (China). Low intra-genus diversity was identified by Krausfeldt et al. (64) in Lake Okeechobee, where only three *Microcystis* MAGs, two of which were annotated as *Microcystis* panniformis (a commonly found species in Lake Taihu)*,* were recovered from a large data set of metagenomes (65). Three out of five of the non-redundant *Microcystis* MAGs in our study were annotated as *M. panniformis*, while the other two MAGs were annotated as *M. aeruginosa* and *Microcystis* sp. (Fig. 5). *M. panniformis* and *M.aeruginosa* are known to share high levels of genomic similarity but differ in their colony morphology, with *M. panniformis* often forming larger colonies, potentially giving them an advantage in shading out their competitors (66). Together, these data show low intra-genus diversity and the common occurrence of *M. panniformis* across subtropical and tropical lakes, indicating a potential ecological pattern across lakes with shared climates (distinct dry and rainy seasons, warm temperatures, high humidity, and reduced seasonality). This data could not be compared directly to other studies on African Great Lakes due to limited data availability, highlighting these high-quality MAGs as a foundational resource for metagenomic research on cyanoHABs in this region.

### BGCs encoding the synthesis of cyanotoxins, cyanopeptides, and unknown metabolites found across the gulf, even in areas without visible cyanoHABs

The diverse makeup of cyanobacteria within the Winam Gulf poses threats not only due to their noxious smells and effects on fishing, tourism, recreation, and wildlife but also due to their ability to produce toxins that are harmful to humans and wildlife (21, 51). We identified the *mcy* BGC responsible for the synthesis of microcystin at over half of the sites and definitively ascribed its source for the first time, solely to *Microcystis*, resolving speculation that *Microcystis* or *Dolichospermum* could harbor these genes (28, 67). The expression of the *mcy* BGC has been shown to increase in response to temperature, presence of reactive oxygen species, light, nitrogen forms and availabilities, and growth rate, though factors that constrain the biosynthesis of microcystins remain convoluted (68, 69). The attribution of microcystin synthesis to *Microcystis* within this tropical, warm, eutrophic basin is important in accurate cyanotoxin human exposure management and prevention, especially in areas where raw water is used for drinking and domestic tasks. A previous study in the Winam Gulf identified high levels of the amidinotransferase gene (*cyrA*) in the cylindrospermopsin-encoding BGC near the Nyando River mouth in June 2022 using quantitative PCR (28, 45). In this study, these genes were not detected at high levels, and our methods were not able to assemble the cylindrospermopsin BGC or link it to an organism.

The bloom-forming cyanobacteria present in the gulf encoded the ability to produce other nitrogen-rich, hepatotoxic cyanopeptides. These BGCs were identified at over half of the sites in the study, including those that did not have a large, visible cyanoHAB event. Many of the sites in this study are heavily used for fishing, aquaculture, bathing, washing clothes, and potable water, underscoring the importance of communicating the dangers of non-visible cyanoHABs to local residents (21, 51). With changing conditions across rainy and dry seasons in the gulf, the composition and expression of these BGCs may change, producing diverse cocktails of harmful molecules beyond microcystins. Such seasonal expression changes were observed in a study in a subtropical river (Florida, USA) where the cyanopeptolin BGC, also identified in our study, was 80 times more expressed than the *mcy* operon in the winter season, while this relationship inverted in the spring season (56). BGC expression studies in tropical regions are needed to begin making hypotheses about metabolites’ complex functional roles and environmental triggers. Moreover, the human health effects of these molecular combinations remain unknown, and BGC expression studies across both tropical and temperate systems indicate the expression of multiple BGCs at any given time (11, 70).

In addition to cyanopeptides and cyanotoxins, cyanobacteria in the gulf encoded over 300 unique BGCs, most of which remain cryptic. These findings illustrate the rich ability of Winam Gulf cyanobacteria to produce uncharacterized metabolites with unknown but potentially harmful or therapeutic properties. The number and diversity of BGCs per MAG in the Winam Gulf were consistent with results from cyanobacteria in Western Lake Erie (37). The diverse biosynthetic repertoire of these bloom-forming cyanobacteria is both an opportunity for discovery and has implications for protecting human health.

### Conclusion

This study characterizes the cyanobacterial assemblage, function, and biosynthetic potential across different regions of the Winam gulf. The metagenomic data revealed different survival strategies available for use by major, bloom-forming cyanobacteria to harness distinct environments and conditions around the gulf, including extensive use of BGCs. Omics techniques (metagenomics, metatranscriptomics, and metabolomics) should be incorporated into current monitoring schemes in the gulf and other African Great Lakes impacted by toxic cyanobacteria to increase research capacity on cyanoHAB ecology and dynamics, inform water management plans, and protect human and ecosystem health. A multi-faceted monitoring plan that incorporates these techniques will look different in various regions depending on the level of cyanoHAB exposure potential and available resources, personnel, and expertise. Moreover, filling this data gap is vital in understanding the biogeographical differences in cyanoHABs, specifically how cyanoHAB events in the global south differ from those in more temperate regions of the world. For example, our results suggest low genomic diversity of dominant cyanobacterial taxa in the gulf, differing from what is observed in temperate regions like the Laurentian Great Lakes but converging with findings from subtropical lakes like Lake Okeechobee and Lake Taihu. Insights from a diverse array of geographic regions will shed valuable insight into how cyanoHABs may respond to a rapidly warming climate across latitudinal gradients.

## MATERIALS AND METHODS

### Data collection

#### Study site and sample collection

This study was conducted on 24–25 June 2022 and 30 May to 3 June 2023 in the Winam Gulf. Thirty-one water samples were collected from a depth of 1 meter using a 2.5 L Van Dorn sampler. Microbial biomass was collected by filtering water (between 60 and 300 mL) through a 0.22 µm Polyethersulfone (PES) Sterivex cartridge filter (MilliporeSigma, Burlington, MA) and preserved in 1.5 mL of Zymo DNA/RNA shield (Zymo, Orange, CA) and placed in a −20°C freezer. Water samples for dissolved nutrient analyses were filtered through a 0.2 µm filter, while unfiltered water was collected for total nutrient analyses and frozen at −20°C. Water was filtered through GF/F filters and stored in a sterile tube for chlorophyll analysis. Nutrient, DNA, and chlorophyll samples were shipped from Kisumu, Kenya at room temperature back to University of Tennessee Knoxville in 2022 and University of Michigan in 2023 for DNA extraction, and Baylor University for nutrient and chlorophyll analyses in 2023. These shipments were accompanied by a letter of support from Kenya Marine and Fisheries Research Institute and/or a United States Department of Agriculture (USDA) permit from David H. Sherman. Physicochemical parameters (i.e., water temperature, dissolved oxygen, conductivity, total dissolved solids, turbidity, and pH) were collected using a multiparameter sonde (YSI Inc., Yellow Springs, OH) on site. Total cyanobacterial abundance was measured using an AlgaeTorch (bbe moldaenke GmnH, Schwentinental, Germany).

#### DNA extraction and sequencing

All sequencing data for this manuscript have been summarized previously in a Microbiology Resource Announcement by Zepernick and Hart et al. (71) and are briefly described below (71). For samples from 2022, DNA was extracted from the Sterivex cartridges after flushing all DNA/RNA shields out of the filter cartridge using phosphate-buffered saline (PBS), prepared according to Cold Spring Harbor’s protocol (72). Duplicate samples from each site in 2022 were collected, extracted, and sequenced. DNA extractions were performed at the University of Tennessee Knoxville using standard phenol-chloroform methods and subsequent ethanol precipitation as reported previously (73). DNA quality was assessed via the Nanodrop ND-100 spectrophotometer (Thermo Fisher Scientific, Waltham, MA) to ensure 260/280 ratios were sufficient (260/280 > 1.80). DNA concentration was quantified using the Qubit double-stranded DNA HS assay kit (Invitrogen, Carlsbad, CA). Extracted DNA was submitted to the University of Minnesota Genomics Core for genomic library construction and sequencing. Sequencing was completed on an Illumina Novaseq system equipped with an S4 flowcell with 150 cycles (150-base pair paired-end reads; Illumina, San Diego, CA).

For samples from 2023, DNA was extracted from the Sterivex cartridges after flushing all DNA/RNA shields out of the filter cartridge using PBS, prepared according to Cold Spring Harbor’s protocol (72). DNA extractions were performed at the University of Michigan. Filter cartridges were opened using a pipe cutter, and the filter membrane was carefully removed using sterile forceps. DNA from the filters was extracted using the DNeasy Powerwater Sterivex Kit (Qiagen, Germantown, MD) according to the manufacturer’s protocol. Quantity was assessed using the high-sensitivity DNA qubit kit (Invitrogen, Carlsbad, CA). Extracted DNA was submitted to the University of Michigan Advanced Genomics Core for genomic library construction and sequencing. Sequencing was completed on an Illumina NovaX system equipped with a 10B flowcell with 300 cycles (150-base pair paired-end reads; Illumina, San Diego, CA).

#### Nutrient analyses

Water samples filtered through Whatman GF/F filters pre-combusted at 550°C for 4 hours were collected in new 50 mL Falcon Tubes and frozen at −20°C until analysis. Filtered dissolved nutrient samples were analyzed for nitrate-nitrogen (NO_3_-N), ammonium-nitrogen (NH_4_-N), and soluble reactive phosphate (PO_4_-P), using Cd reduction, phenate, and molybdate methods, respectively (74). Total dissolved nitrogen and total dissolved phosphorus were analyzed from filtered samples using the Cd reduction and molybdate methods (75). Unfiltered samples were analyzed for TN and TP using the Cd reduction and molybdate methods (75). All soluble and total nutrients were analyzed using a Lachat QuickChem 8500 Series II autoanalyzer (Lachat Instruments, Hach Company) at the Center for Reservoir and Aquatic Systems Research located at Baylor University, Waco, TX. Chlorophyll a in extracts was measured using the non-acidification method of Welschmeyer on a Turner Designs Trilogy fluorometer calibrated with pure Chlorophyll a standards (Turner Designs, Sunnyvale, CA, USA) (76).

### Bioinformatic analysis

#### Metagenomic data processing

Metagenomes were processed with the Great Lakes Atlas for Multi-Omics Research (GLAMR) pipeline (GLAMR Portal, GLAMR Github). fastp v0.23.2 was used to deduplicate (--dup_calc_accuracy 6), remove adapters (--detect_adapter_for_pe), trim (--cut_front --cut_tail --cut_window_size = 4—cut_mean_quality --cut_mean_quality 20), and filter (--length_required 50 n_base_limit 5—low_complexity_filter --complexity_threshold 7) raw sequence reads (77). BBmap was used to remove human contaminant reads mapping to the GENCODE release 38 human genome (78). Each sample was assembled using Megahit v1.2.9 with the meta-sensitive parameter (79). Assembled contigs longer than 2,000 base pairs were used for binning. Concoct v1.1.0, MaxBin2 v2.2.7, Metabat2 v2.17, MetaDecoder v1.0.13, SemiBin v1.0.3, and VAMB v3.0.8 were used to bin contigs into MAGs (80–85). MAGs were clustered and dereplicated at 98% identity using dRep v3.2.0 (86, 87). MAG quality was assessed with CheckM v.1.2.2, and MAG taxonomy was assigned using GTDBtk and the GTDB release 214 database (40, 88).

#### Community composition analysis

MAGs from all 31 samples were grouped and dereplicated using dRep v3.2.0 at 98% (87). MAG quality statistics, calculated with checkM v1.2.2 and bakta v1.8.1, can be found in Table S2 (88, 89). Quality-controlled (QC) reads were mapped to the dereplicated set of MAGs using CoverM v0.6.1 (using the default mapper: MiniMap2 v2.24) to get the relative abundance of each MAG in each sample (Fig. 3) (90, 91). Observations from duplicate samples from 2022 were averaged in all applicable metagenomic methods in the study due to the replicate’s similarity (Fig. S5). A Bray-Curtis dissimilarity matrix was calculated from the relative abundance of all cyanobacterial genera in each sample and assessed using NMDS using the vegan v2.6 package in the R statistical software (92). Cyanobacterial makeup was assessed in relation to environmental parameters using the envfit function in the vegan v2.6 package in the R statistical software (Fig. 4A) (92). The correlation of the six most abundant genera with each other and associated environmental parameters was assessed using Spearman’s correlation coefficient in the R statistical software.

#### Phylogenomic analysis

High-quality MAGs (checkM: ≥90% completeness and ≤10% contamination) annotated by GTDBtk as *Microcystis*, *Dolichospermum*, *Planktothrix*, and *Sphaerospermopsis* were dereplicated at 99.5% identity with dRep v3.2.0 to produce representative bins for each of these genera (40, 87, 88). A tree was built using these MAGs and reference genomes from NCBI (Table S6) using GToTree v1.7.05 (93, 94). A maximum likelihood tree for each taxa using 251 marker genes was generated. Reference genomes for the *Microcystis* (Fig. 5A), ADA (Fig. 5B), and *Planktothrix* (Fig. 5C) trees were sourced from prior studies (36, 37, 63, 95, 96). Reference genomes for the *Spaherospermopsis* tree (Fig. 5D) were chosen from the NCBI genome database if they were characterized as either a reference genome or a strain (93). An outgroup represented by *Cyanobium gracile PCC 6307* (NCBI: PRJNA158695) was used in each of the four trees. Phylogenomic trees were visualized in Interactive Tree of Life (ITOL) (97).

#### Nitrogen fixation genomic potential detection

QC reads from each sample were mapped to the *nif*H, *nif*D, *nif*K, *Chl*N, and *Chl*B databases in NFixDB (accessed March 2024, Database) using MiniMap2 v2.24 (43, 91). Hits to *Chl*N and *Chl*B were excluded but were used in the read mapping step to ensure a competitive database was used (98). Reads that mapped to the reference with 80% coverage and 90% identity were kept. Hits to *nif*H, *nif*D, and *nif*K were kept if 80% of the gene was covered with 90% identity (Fig. 6). *nif*HDK was used to ensure the identification of true nitrogen fixers and not pseudo-*nif*H-containing microbes (42). Total fragments per kilobase per million mapped reads (FPKM) was calculated for each gene in each sample using Equation 1 (99).


(1) $$\begin{array}{rl} & \text{ Total Fragments per Kilobase per Million Mapped Reads (FPKM) }\\ & =\frac{\text{ total no. of mapped reads }}{\left(\text{ length of nif gene }/1,000)\times (\text{ total no. of reads per sample }\right)}\times 1,000,000 \end{array}$$

#### Cyanopeptide and cyanotoxin BGC detection

QC reads from each sample were mapped to all BGCs in the MIBiG database from the cyanobacterial genera *Microcystis*, *Dolichospermum*, *Planktothrix*, *Sphaerospermopsis*, *Anabaena*, *Aphanizomenon*, *Cylindrospermopsis*, and *Cyanobium* using MiniMap2 v2.24 (44, 91). Reads that mapped to the reference with 80% coverage and 90% identity were kept. Hits to BGCs were kept if 70% of the BGC was covered by reads in that sample. FPKM was calculated for each BGC in each sample using Equation 2 (99).


(2) $$\begin{array}{rl} & \text{ Total Fragments per Kilobase per Million Mapped Reads (FPKM) }\\ & =\frac{\text{ total no. of mapped reads }}{\left(\text{ length of BGC }/1,000)\times (\text{ total no. of reads per sample }\right)}\times 1,000,000 \end{array}$$

#### Mcy and cyr operon identification and comparison to references

All assemblies in the data set were searched for the presence of genes from the *mcy* (MiBIG BGC: BGC0001016) and *cyr* (MiBIG BGC: BGC0000978) operons via BLASTn (44, 100). If genes from either of these operons were present in the assembly of a sample, the sample’s MAGs were analyzed for all genes from the operons using BLASTn. Hits to genes of interest were kept if they had 80% identity and coverage in the queried MAG. MAGs with multiple genes from the reference operon present were analyzed in Anvi’o to identify if the whole reference operon was present and if it was correctly assembled and binned into the MAG (101). The *cyr* operon could not be recovered from this data set, but low levels of the core biosynthetic genes of the *cyr* operon (MiBIG BGC: BGC0000978) were detected via BLASTn at Site 27. Bridge Island (Fig. S1) (100). The *mcy* operon was detected in multiple MAGs, all identified as *Microcystis* by GTDB-Tk (40). One MAG from Site 1. Homa Bay (NCBI Accession: SAMN41711332) was chosen for MAG analysis in Anvi’o to confirm the taxonomic identity of the contig with the *mcy* operon (Fig. S3). Additionally, the *mcy* operon detected in the MAG was compared to the *mcy* operon from *Microcystis aeruginosa* FACHB 1326 (GenBank: OQ291092.1) using the program “clinker” (Fig. S3) (102).

#### Biosynthetic gene cluster mining and abundance analysis

BGCs were mined from medium- and high-quality cyanobacterial MAGs (≥70% completion and ≤30% contamination) using antiSMASH v7.0 (46). All BGCs greater than 5,000 base pairs long were extracted and dereplicated at 99% using dRep v3.2.0 (Table S6) (87). Gene clusters were grouped into GCFs using BiG-SCAPE and visualized in cytoscape v3.9.1 ((Fig. S1) (103, 104). QC reads from each sample were mapped to the dereplicated set of BGCs using MiniMap2 v2.24 (91). Reads that mapped to the reference with 80% coverage and 90% identity were kept. Hits to BGCs were kept if 70% of the BGC was covered by reads in that sample (Fig. 9). FPKM for each BGC was calculated using Equation 2. Total FPKM for each BGC type (x-axis) was calculated by summing the FPKM of each BGC belonging to that BGC type per sample.

#### Statistical analysis and figure rendering

All statistical analysis and plots were created using R and R studio v4.3.3 (105). The R packages vegan, tidyverse, and RColorBrewer were used for figure creation (92, 106, 107).

## Data Availability

Raw sequences are available on the NCBI SRA under BioProject PRJNA1119566 and BioSamples SAMN41659670- SAMN41659717. All MAGs have been deposited in the NCBI database under BioProject PRJNA1119566 and BioSamples SAMN41711301-SAMN41712291. Physicochemical data are available on the Biological and Chemical Oceanography Data Management Office (BCO-DMO) site (108). All code used in the analysis and visualization of the data presented in this study can be found on GitHub.

## AFTER EPUB

*[This article was published on 8 January 2025 with the Importance section missing. The Importance was added in the current version, posted on 6 February 2025.]*

## SUPPLEMENTAL MATERIAL

Fig. S1 aem.01507-24-s0001.eps Read mapping (via blastn) from Site 27.

Fig. S2 aem.01507-24-s0002.eps Gene cluster families (GCFs) for identified BGCs via antiSMASH and BiG-SCAPE.

Fig. S3 aem.01507-24-s0003.eps Genome profile of MAG samp_4317_maxbin_26, annotated as *Microcystis* sp. (NCBI: SAMN41711332).

Fig. S4 aem.01507-24-s0004.eps Biosynthetic potential composition in relation to dominant cyanobacterial genera relative abundances in the Winam Gulf.

Fig. S5 aem.01507-24-s0005.eps Microbial and archaeal composition makeup in Winam Gulf, LV 2022 metagenome replicates.

Supplemental legends aem.01507-24-s0006.pdf Supplemental legends, tables, and figures.

Supplemental tables aem.01507-24-s0007.xlsx Tables S1 to S6.

ASM does not own the copyrights to Supplemental Material that may be linked to, or accessed through, an article. The authors have granted ASM a non-exclusive, world-wide license to publish the Supplemental Material files. Please contact the corresponding author directly for reuse.
