# Supplementary material for: Metagenomics reveals spatial variation in cyanobacterial composition, function, and biosynthetic potential in the Winam Gulf, Lake Victoria, Kenya
Source: Appl Environ Microbiol. 2025 Jan 8;91(2):e01507-24. doi: 10.1128/aem.01507-24 (PMC11837572; doi:10.1128/aem.01507-24)
Supplement: Supplemental legends — , tables, and figures. [file aem.01507-24-s0006.pdf]

**Supplementary Material**

**Supplementary tables: Found attached in .xlsx file.**

- **Table S1:** Table of all metadata for sampled sites in study
- **Table S2:** Table of genome statistics for 98 percent dereplicated set of MAGs
- **Table S3:** Table of spearman correlation coefficients between highly abundant cyanobacterial genera and associated metadata variables.
- **Table S4:** Table of genome statistics for high quality cyanobacterial MAGs.
- **Table S5:** Manual annotation notes for dereplicated set of BGCs identified from high quality cyanobacterial MAGs from the sampled sites in Winam Gulf.
- **Table S6:** Reference genomes used for phylogenomic analysis in Figure 5 of manuscript.

**Supplementary Figures:**

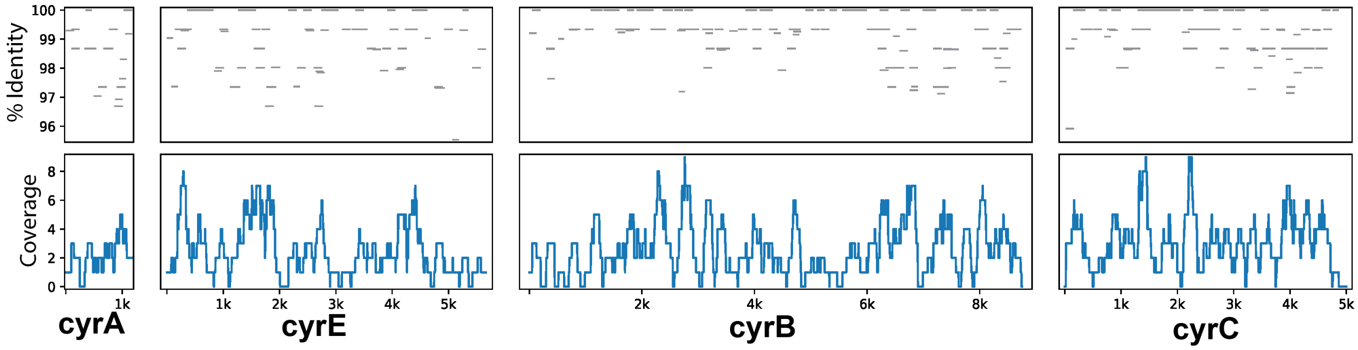


**Figure S1**: Read mapping (via blastn) from Site 27. Bridge Island to four genes from the cylindrospermopsin BGC from *Cylindrospermopsis raciborskii AWT205* (MIBiG BGC: BGC0000978). Percent identity of reads mapped to these genes is shown in the top quadrants, and percent coverage by mapped reads to these genes is shown in the bottom quadrant.

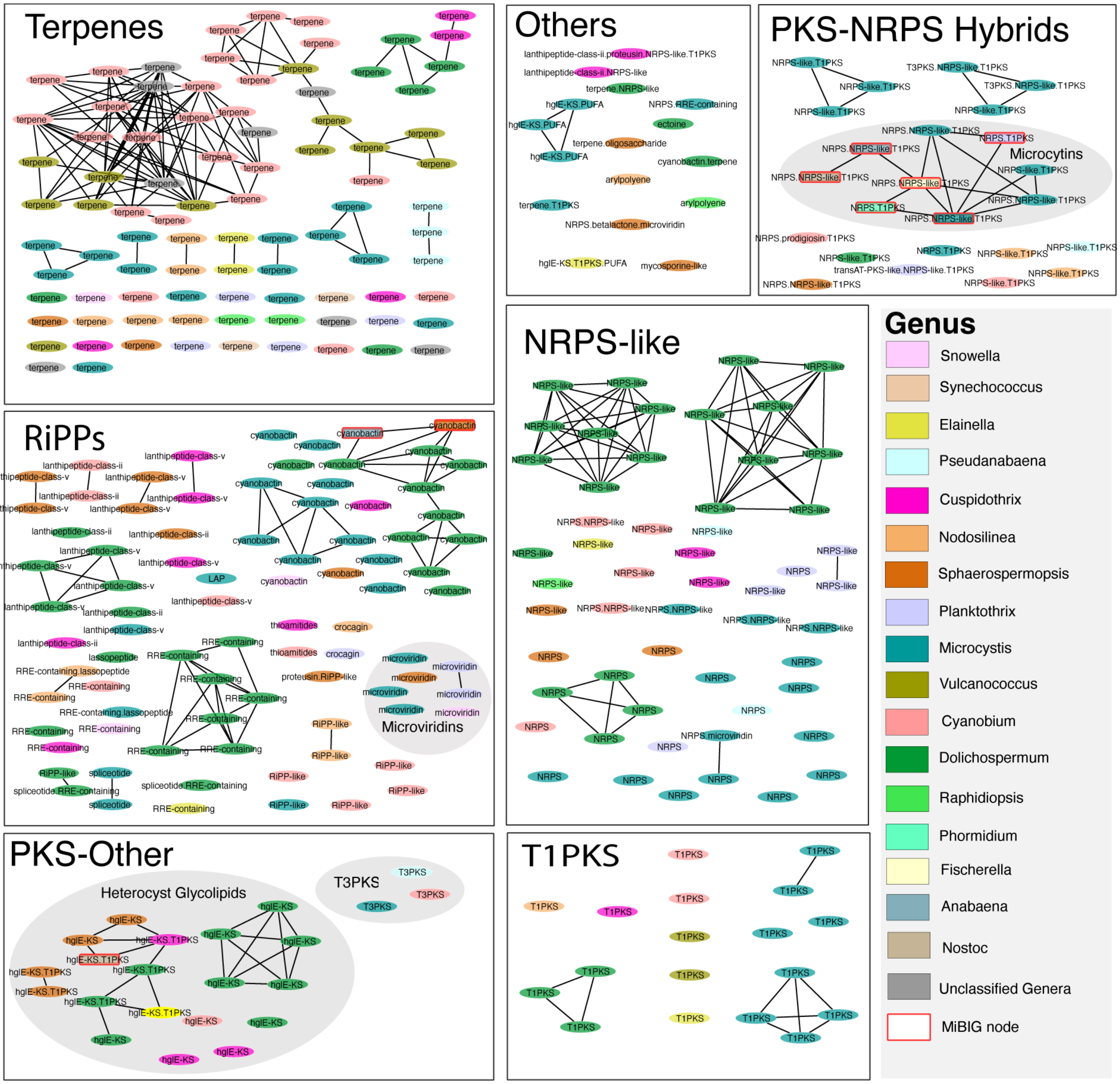


**Figure S2:** Gene cluster families (GCFs) for identified BGCs via antiSMASH and BiG-SCAPE. BGCs identified from high quality cyanobacterial MAGs via antiSMASH v7 were manually annotated (SI Table 2) and analyzed via BiG-SCAPE. Nodes are colored based on the cyanobacterial genus of the MAG they originate from (based on GTDB-Tk taxonomic identification). Nodes are labeled based on manual annotation of BGCs. Square nodes with a red outline are from the MiBIG database, linking BGCs to known biosynthesis products. Edges between nodes indicate similarity of BGCs calculated through a distance matrix that considers number of shared PFAM domains, pairs of adjacent PFAM domains, and sequence similarities between protein sequences. Gray circles around nodes indicate the putative synthesis product of those nodes based on antiSMASH annotation and manual inspection of core and additional biosynthesis genes.

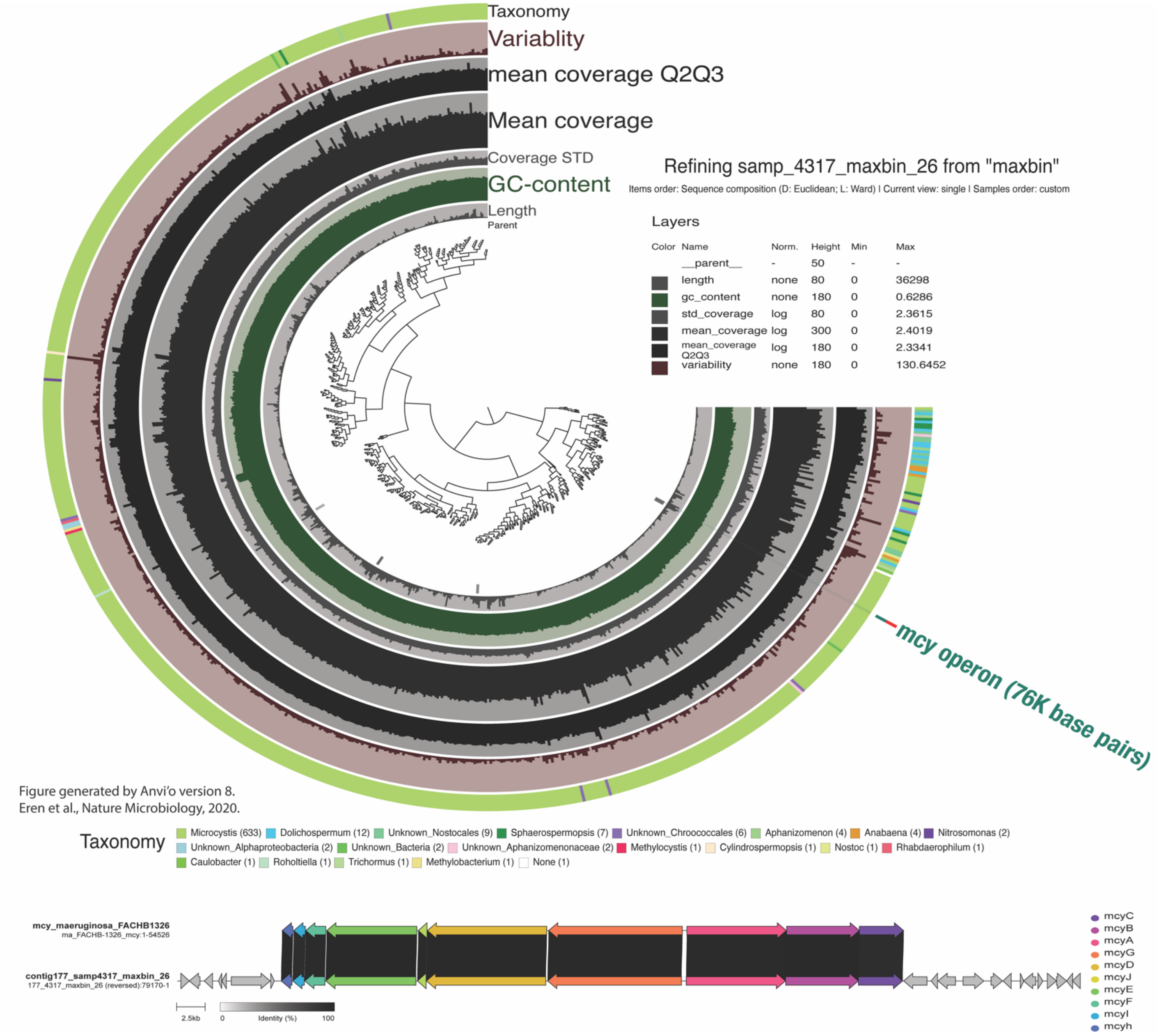


**Figure S3:** Genome profile of MAG samp_4317_maxbin_26, annotated as *Microcystis* sp. (NCBI: SAMN41711332). The contig with the *mcy* operon is indicated in the Anvi'o MAG profile. Gene similarity image from clinker shows the reference *mcy* operon from *Microcystis aeruginosa* FACHB-1326 (GenBank: OQ291092.1) and its close similarity to the *mcy* operon identified in samp_4317_maxbin_26 on contig 177 below.

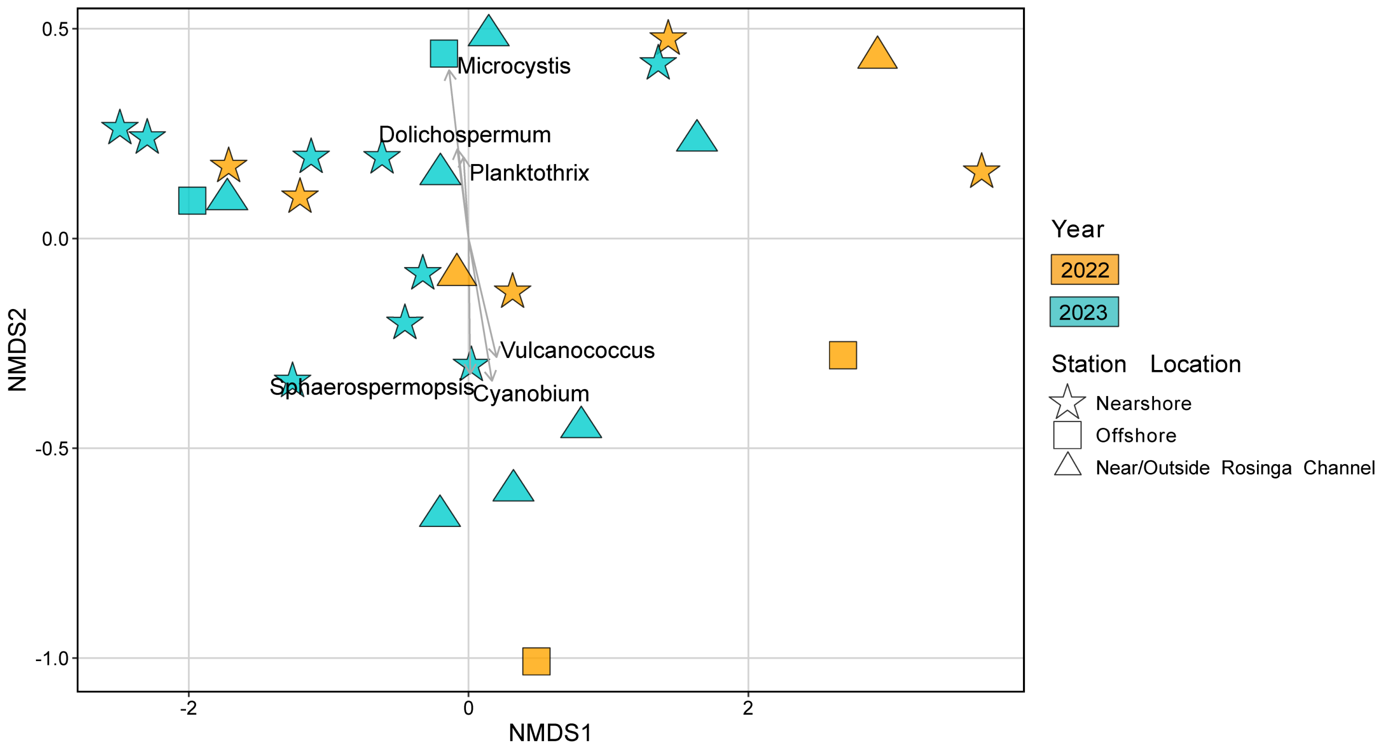


**Figure S4:** Biosynthetic potential composition in relation to dominant cyanobacterial genera relative abundances in the Winam Gulf. NMDS analysis of the relative abundance of all identified BGCs from each sample site. Arrows and labels represent the direction and strength of the relationship between the ordination axes and the relative abundance of the top 6 most abundant cyanobacterial genera across the samples. Station location is indicated by the shape of ordination points, and samples from 2022 are represented in amber, while samples from 2023 are in teal. Samples taken from rivers (n=4) were excluded from this ordination because no BGCs were identified in them.

A.

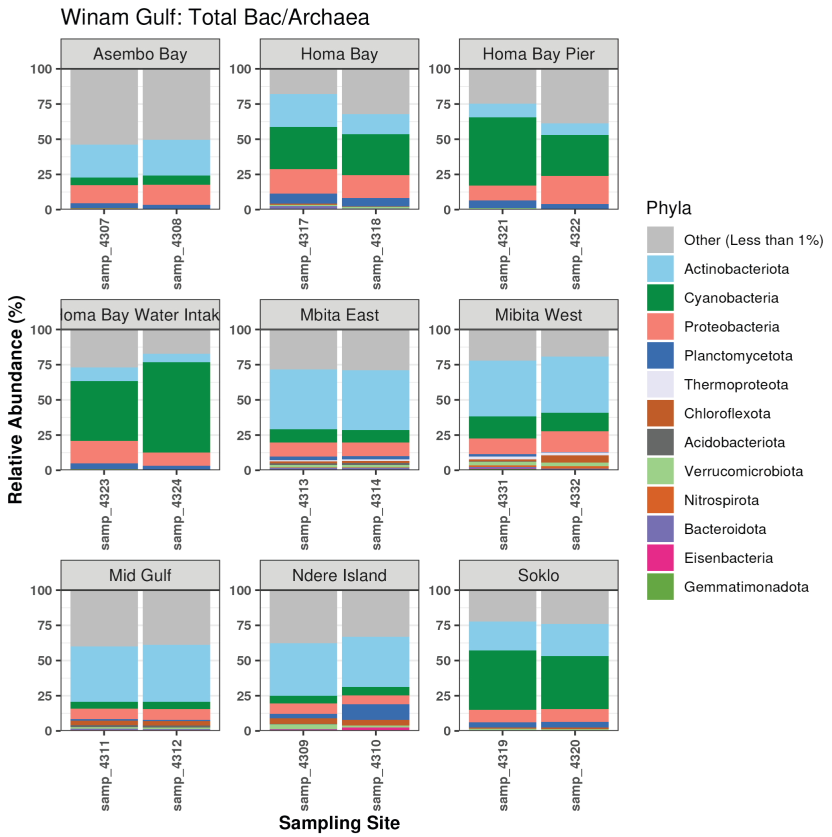
Winam Gulf: Total Bac/Archaea
Asembo Bay
Homa Bay
Homa Bay Pier
loma Bay Water Intak
Mbita East
Mibita West
Mid Gulf
Ndere Island
Soklo
Relative Abundance (%)
Sampling Site
samp_4307
samp_4308
samp_4317
samp_4318
samp_4321
samp_4322
samp_4323
samp_4324
samp_4313
samp_4314
samp_4331
samp_4332
samp_4311
samp_4312
samp_4309
samp_4310
samp_4319
samp_4320
Phyla
Other (Less than 1%)
Actinobacteriota
Cyanobacteria
Proteobacteria
Planctomycetota
Thermoproteota
Chloroflexota
Acidobacteriota
Verrucomicrobiota
Nitrospirota
Bacteroidota
Eisenbacteria
Gemmatimonadota

B.

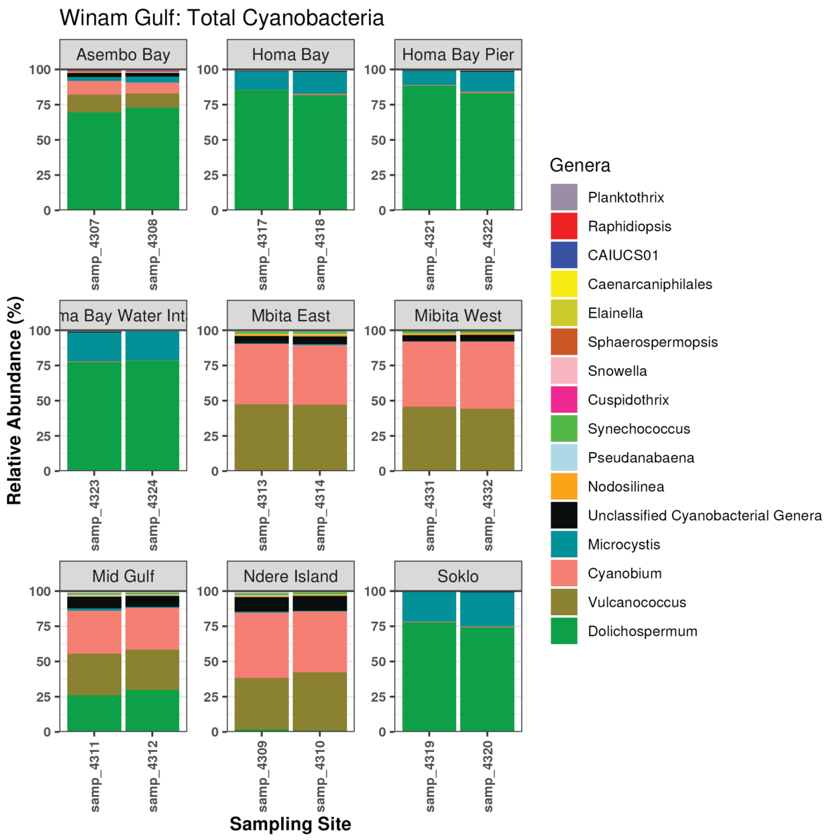
Winam Gulf: Total Cyanobacteria
Asembo Bay
Homa Bay
Homa Bay Pier
ma Bay Water Int
Mbita East
Mibita West
Mid Gulf
Ndere Island
Soklo
Relative Abundance (%)
Sampling Site
samp_4307
samp_4308
samp_4317
samp_4318
samp_4321
samp_4322
samp_4323
samp_4324
samp_4313
samp_4314
samp_4331
samp_4332
samp_4311
samp_4312
samp_4309
samp_4310
samp_4319
samp_4320
Genera
Planktothrix
Raphidiopsis
CAIUCS01
Caenarcaniphilales
Elainella
Sphaerospermopsis
Snowella
Cuspidothrix
Synechococcus
Pseudanabaena
Nodosilinea
Unclassified Cyanobacterial Genera
Microcystis
Cyanobium
Vulcanococcus
Dolichospermum

**Figure S5:** Microbial and archaeal composition makeup in Winam Gulf, LV. (A) Relative abundance (percentage) of bacterial and archaeal phyla from 2022 samples with replicates is shown. The phyla, annotated with GTDB-Tk release 214, representing less than 1 percent of the population were grouped in the "Other" category (87). (B) Relative abundance (percentage) of cyanobacterial genera from 2022 samples with replicates is shown. Cyanobacteria with no genus taxonomic assignment from GTDB-Tk were labeled as "unclassified cyanobacteria". Unmapped reads were excluded from this analysis and mapped reads were normalized to 100 percent. Metagenomic samples from the same sampling site are grouped together in a facet.
